# Ewe Vaginal Microbiota: Associations With Pregnancy Outcome and Changes During Gestation

**DOI:** 10.3389/fmicb.2021.745884

**Published:** 2021-10-22

**Authors:** Lucas R. Koester, Amy L. Petry, Curtis R. Youngs, Stephan Schmitz-Esser

**Affiliations:** ^1^Department of Veterinary Microbiology and Preventive Medicine, Iowa State University, Ames, IA, United States; ^2^Interdepartmental Microbiology Graduate Program, Iowa State University, Ames, IA, United States; ^3^Department of Animal and Food Sciences, Texas Tech University, Lubbock, TX, United States; ^4^Department of Animal Science, Iowa State University, Ames, IA, United States

**Keywords:** ovine, sheep, fertility, reproduction, vaginal microbiome

## Abstract

Reproductive performance is paramount to the success of livestock production enterprises focused on lamb meat production. Reproductive success is influenced by various factors, possibly including the reproductive tract microbial communities present at the time of copulation and throughout pregnancy. There are few publications that identify the vaginal microbial communities of livestock, and even fewer exist for sheep. To compare ewe vaginal microbial communities, vaginal swabs were taken from 67 Hampshire and Hampshire X Suffolk crossbred ewes from the Iowa State University sheep farm at a pre-breeding time point (S1) and after pregnancy testing (S2). Animals that were determined pregnant were sampled again within a few days of expected parturition (S3). DNA was extracted from these swabs, and 16S rRNA gene Illumina MiSeq amplicon sequencing was conducted to fingerprint the bacterial communities found within this system. Pre-breeding time point samples showed no differences in community structure between animals later found to be pregnant or non-pregnant, but significant changes were detected in species richness (Chao; *P* < 0.001) and species diversity (Shannon; *P* < 0.001) at the second sampling time point. A higher microbial diversity within the S2 time point samples may suggest a more stable environment driven by pregnancy, as this increased diversity is maintained in pregnant animals from the S2 to the S3 time point. Additionally, several bacterial phylotypes, such as *Mannheimia*, *Oscillospiraceae*-like OTUs and *Alistipes*, were more abundant at either the S1 or S2 time points in animals that established pregnancy, suggesting a beneficial effect on pregnancy outcome. This study identifies changes within the microbial communities of the ewe vagina before and during gestation and offers inferences on how these changes may impact pregnancy outcome. Information presented herein offers new knowledge about sheep vaginal microbial communities and serves as a starting point to help guide researchers to improve sheep reproductive performance in the future.

## Introduction

Reproductive success is an important determinant of profitability for commercial sheep production enterprises focused on lamb meat production (Amer et al., 1999; Newton et al., 2017), and understanding factors that influence establishment of pregnancy in the ewe is key to improving flock reproductive performance. Although numerous studies have shown the impact of factors such as age and nutrition on ewe reproductive performance (Gaskins et al., 2005; Pettigrew et al., 2019), there is a paucity of information regarding if and how the microbiota of the ewe reproductive tract may contribute to pregnancy outcome.

Fluctuations within the host-associated microbial populations can explain specific phenotypes within a population. Shifts in microbial populations can potentially be used as biomarkers when attempting to identify a specific phenotype, even before the phenotype is clearly defined. It is well known that the microbiota within the gastrointestinal tract of the ruminant is linked to several important aspects of host nutrition, metabolism, and health (Yeoman and White, 2014; Shabat et al., 2016; O’Hara et al., 2020). Other than knowledge about specific reproductive tract pathogens, comparatively little information is available about how the microbial communities as a whole, or specific members of the microbial communities within the vaginal tract, may influence aspects of reproduction in livestock.

In beef cattle, the vaginal microbiota has been associated with pregnancy outcome (Laguardia-Nascimento et al., 2015; Deng et al., 2019). Similarly, the vaginal microbiota of dairy cattle has been correlated with periparturient reproductive problems (Bicalho et al., 2017). In the pig, microbial populations present in the vagina were also associated with reproductive outcomes (Sanglard et al., 2020a,b).

Bacteria may indirectly modify the reproductive tract environment through alterations in pH, sperm adherence, and by stimulating an inflammatory response (Dai et al., 2015; Zhou et al., 2015; Santos-Greatti et al., 2016; Wang et al., 2020). Some of these factors, especially pH, can directly affect important aspects of reproduction such as sperm motility, fertilization rate, and early embryonic development and survival (Wang et al., 2020; Ravel et al., 2021). Only a handful of studies have offered insights into the vaginal microbial communities within ruminants (Swartz et al., 2014; Laguardia-Nascimento et al., 2015; Deng et al., 2019; Messman et al., 2020) and associated those findings with different reproductive outcomes (Swartz et al., 2014; Deng et al., 2019; Messman et al., 2020). Compared with other ruminants, knowledge about the microbial communities in the reproductive tract of sheep is even less. A few cultivation-based studies have provided initial characterization of the ewe reproductive tract microbiota (Sawyer, 1977; Gatti et al., 2011; Manes et al., 2018). However, to the authors’ knowledge, only two studies have performed 16S rRNA gene-based amplicon sequencing of sheep vaginal microbial communities (Swartz et al., 2014; Serrano et al., 2020).

The objectives of this study were to: (1) characterize the microbial communities present in the vagina of sheep using 16S rRNA gene targeted amplicon sequencing, (2) investigate potential influences of the vaginal microbiota on pregnancy outcome, and (3) examine the stability of the vaginal microbial community throughout mid- to late-gestation in pregnant ewes.

## Materials and Methods

### Ethics Statement

All animal procedures in this study were conducted after approval by the Iowa State University Institutional Animal Care and Use Committee (protocol no. 18-206).

### Experimental Animals and Sample Collection

A total of 67 Hampshire (*n* = 44) and Hampshire × Suffolk crossbred (*n* = 23) ewes from the Iowa State University sheep farm were utilized for this study. Ewes were maintained in a drylot and fed a diet consisting of orchardgrass hay (*Dactylis glomerata L.*) and shelled corn. The first vaginal swab was collected from each female in the pre-breeding period [designated as sample 1 (S1)], immediately prior to insertion of a controlled internal drug-releasing device (CIDR) containing 0.3 g progesterone for synchronization of estrus. At the time of CIDR removal, ewes were segregated into one of four different single-sire drylot mating pens in accordance with farm genetic management plans. After the end of the 51-day breeding period, breeding males were removed and ewes were co-mingled until the time of pregnancy testing approximately 40 days after rams were removed from mating groups.

Real-time B-mode ultrasound was used to determine pregnancy outcome (pregnant, non-pregnant) and to estimate fetal age. A vaginal swab was collected from all ewes after pregnancy testing, and samples collected at this time point were designated as S2. Non-pregnant ewes were culled from the flock and therefore were no longer available for subsequent sampling. A pre-lambing vaginal swab collection (designated as S3) was scheduled for all pregnant ewes within 7 days of expected lambing based on ultrasound-estimated fetal age and an expected gestation length of 147 days. However, ten pregnant ewes lambed earlier than predicted and, as a consequence, S3 samples were not collected from all pregnant ewes.

To collect the ewe vaginal microbial community, vaginal swabs were obtained using a sterile 17.8 cm histology brush (Puritan Medical Products) inserted approximately 8 cm into the vagina. The labia were parted manually by a gloved sampling technician to ensure the brush never contacted any external surface. After insertion into the vagina, the brush was gently spun three times against the vaginal wall. The brush was carefully removed from the vagina ensuring no contact with any surfaces to avoid contamination and was subsequently placed inside a sterile 15-ml conical tube containing 10 ml of sterile 1x phosphate buffered saline (PBS). The histology brush was cut using a sterilized cutting tool to enable the terminal portion of the brush to be wholly contained within the tube and completely submerged in sterile PBS. The cap on each tube was tightly closed before being immediately placed on ice for transport to the laboratory where samples were stored at −80°C until DNA extraction was performed.

### Sample Processing

To extract DNA from the vaginal samples, the tubes containing swabs in PBS were thawed in a 37°C water bath and vigorously vortexed for 15 min to detach the cells from the histology brushes. After removal of the brush from the tube, the remaining material suspended in the PBS was spun for 5 min at 4,694 × *g* using a centrifuge (Thermo Scientific, Legend XR1). The supernatant was removed from the tube, and the pellet was resuspended in 750 μL of the PowerLyzer solution from the DNeasy PowerLyzer Powersoil kit (Qiagen, Germantown, MD, United States). DNA was subsequently extracted following the manufacturer’s instructions. Mechanical cell lysis was performed using a Fisher Scientific Bead Mill 24, and DNA concentrations were determined using a Qubit 3 fluorometer (Invitrogen, Carlsbad, CA, United States).

After DNA extraction, samples were diluted with sterile 0.1% diethylpyrocarbonate (DEPC) water to attain a concentration of 25 ng DNA/μl. Sequencing of DNA was performed at the Iowa State University DNA facility using the Illumina MiSeq platform (Illumina, San Diego, CA, United States). Briefly, the genomic DNA from each sample was amplified using Platinum^TM^ Taq DNA Polymerase (Thermo Fisher Scientific, Waltham, MA, United States) with one replicate per sample using universal 16S rRNA gene bacterial primers {515F [5′-G TGYCAGCMGCCGCGGTAA-3′; (Parada et al., 2016)] and 806R [5′-GGACTACNVGGGTWTCTAAT-3′; (Apprill et al., 2015)]}, amplifying the hypervariable region V4 as previously described (Kozich et al., 2013). All samples underwent PCR with an initial denaturation step at 94°C for 3 min, followed by 45 s of denaturation at 94°C, 20 s of annealing at 50°C, and extension for 90 s at 72°C. This process was repeated for 35 total PCR cycles; PCR was finished with a 10-min extension at 72°C. All PCR products were then purified with the QIAquick 96 PCR Purification Kit (Qiagen, Hilden, Germany) according to the manufacturer’s instructions. The PCR bar-coded amplicons were mixed at equal molar ratios and used for Illumina MiSeq paired-end sequencing with a 250-bp read length and cluster generation with 10% PhiX control DNA.

### Sequence Analysis

Sequence analysis was performed using Mothur V1.43.0 software following the Mothur MiSeq Standard Operating Procedure (Kozich et al., 2013). A minimum sequence length threshold of 252 bases was selected based on the bottom 2.5 percentile, and barcode sequences, primers and low-quality sequences were trimmed using a minimum average quality score of 35, with a sliding window size of 50 bp. Sequences with any ambiguous base calls or homopolymers exceeding eight bases were removed as well. Chimeric sequences were removed with the “Chimera.vsearch” command. For alignment and taxonomic classification of operational taxonomic units (OTUs), the SILVA SSU NR reference database (V138) provided by the Mothur website was used. Sequences were clustered into OTUs with a cutoff of 99% 16S rRNA sequence similarity (=0.01 distance). In addition to the SILVA classification, representative sequences of the 50 most abundant OTUs were assigned additional taxonomic information using BlastN against NCBI NR. To reduce the number of spurious OTUs, all OTUs represented by less than 10 reads were deleted. All samples were analyzed together to keep OTUs consistent across experimental questions. Three samples were removed from the analysis due to insufficient read depth, and the remaining 174 samples were randomly subsampled to 8,000 reads using Mothur to accommodate the sample with the lowest number of reads across data sets.

### Statistics

Data considering pregnancy status were analyzed with a compound symmetry dependent covariance structure according to the following statistical model:


(1) $$Y_{i\,j\,k}=\mu +\tau_{i}+\upsilon_{j}+\tau_{i}\,\upsilon_{j}+e_{i\,j\,k}$$

Where Y_*ijk*_ is the observed value for k^th^ experimental unit within the i^th^ level of pregnancy status (non-pregnant vs. pregnant) at the j^th^ sampling (S1 vs. S2); μ is the general mean; τ_*i *_ is the fixed effect of the i^th^ pregnancy status (i = non-pregnant vs. pregnant); υ_*j*_ is the repeated fixed effect of the j^th^ sampling (j = S1 vs. S2); τ_*i*_υ_*j*_ is the subsequent interaction of sampling and pregnancy status; and *e*_*ijk*_ is the associated variance as described by the model for *Y*_*ijk*_ (k = 1 through 67). Because each animal was sampled more than once (S1 and S2), animal was included as a repeated measures random effect to account for the co-variance among samples. Different covariance structures were selected for each test compound symmetry (CS), unstructured (UN), or heterogeneous first-order autoregressive structure [AR(1)] based on lowest BIC values (lowest values represent best fit).

Within gestating ewes, the influence of time on microbial composition were analyzed according to the following reduced statistical model:


(2) $$Y_{i\,j}=\mu +\upsilon_{i}+e_{i\,j}$$

Where *Y*_*ij*_ is the observed value for j^th^ experimental unit at the i^th^ sampling (i = S1, S2, or S3); and *e*_*ij*_ is the associated variance as described by the model for *Y*_*ij*_ (j = 1 through 54). Animal was implemented as repeated effect within the reduced model, incorporating the aforementioned covariance structures based on lowest BIC criteria.

Measurements of Chao species richness, Shannon Diversity, and Simpson evenness were taken to compare community structures between experimental groups. The means of the experimental group alpha diversity measures were analyzed using the PROC MIXED procedure according to the described model or a reduced model when the interaction term was not appropriate. Additionally, Bray-Curtis dissimilarity between experimental groups were analyzed using the Adonis command (PERMANOVA) and BetaDisper command (BetaDisperser) provided within the VEGAN [v2.5-5; (Oksanen et al., 2019)] package according to these same models. Overall variation in bacterial communities was visualized using principle coordinate analysis (PCoA). Canonical analysis of principle coordinates [CAP; (Anderson and Willis, 2003)] was used to visualize the variation based on the models proposed above. This information was generated with Phyloseq [v1.34.0; (McMurdie and Holmes, 2013)] and Vegan. All plotting was completed using ggplot2, v2_3.1.1 graphing package in R 4.1.0.

Additionally, the absolute abundance of the 100 most abundant OTUs among samples and the relative abundance of classified genera and phyla were analyzed using a negative binomial distribution in GLIMMIX procedure of SAS (Version 9.4, SAS Inst., Cary, NC, United States) according to the models described above. All count data were offset by the total library count for a given sample. Corresponding *P*-values were corrected for false discovery rates using the MULTITEST procedure of SAS. Least square means were separated using Fisher’s Least Significant Difference test, and treatment differences were considered significant if *P* or *Q* values were <0.05. For the top 100 OTUs, genera, and phyla classifications with a treatment *Q* value of <0.05, the log_2_-fold change was calculated comparing pregnant and non-pregnant animal groups at time points S1 and S2 and pregnant animals at time points S1, S2, and S3.

### Data Availability

The 16S rRNA gene sequences have been submitted to the NCBI Sequence Read Archive SRA and are available under the BioProject ID PRJNA739687.

## Results

Of the 67 ewes exposed to males for breeding, 53 were pregnant on the day of ultrasonographic pregnancy testing and 14 were not pregnant. Thus, two distinct phenotypes were available for comparison: pregnant and non-pregnant.

### Overview of the Entire Dataset

When considering the entire dataset, 11,026 OTUs were generated from 174 samples after quality control and removal of OTUs representing less than 10 sequences. The average sequencing depth per sample was 25,428 sequences with a standard deviation of 7,600 sequences. Of those reads, 98.1% were bacterial and 1.8% were archaeal. The 11,026 OTUs were assigned to 32 phyla with *Firmicutes, Proteobacteria*, and *Bacteroidetes* being the three most abundant phyla representing 52, 16, and 11% of all reads, respectively (Supplementary Table 1).

Some notable bacterial families that were present within the dataset included *Pasteurellaceae*, *Oscillospiraceae*, *Leptotrichiaceae*, *Staphylococcaceae*, *Lachnospiraceae*, and *Campylobacteraceae* representing 8.9, 6.2, 5.9, 5.6, 5.2, and 2.3% of all reads from the entire data set. The most abundant OTU (OTU 1) was classified as *Ureaplasma* and accounted for 9.9% of all reads. Other prevalent OTUs were OTU 2 (Unclassified *Pasteurellaceae*), OTU 3 (Unclassified *Leptotrichiaceae*), OTU 4 (*Escherichia-Shigella*), and OTU 5 (*Histophilus*) which accounted for 4.9, 4.0, 2.7, 2.5, and 2.1% of the reads, respectively. A list of the 50 most abundant sheep vaginal OTUs can be found in Supplementary Table 2.

### Retrospective Comparison of Pre-breeding (S1) Samples in Ewes Detected as Pregnant or Non-pregnant

**FIGURE 1:**
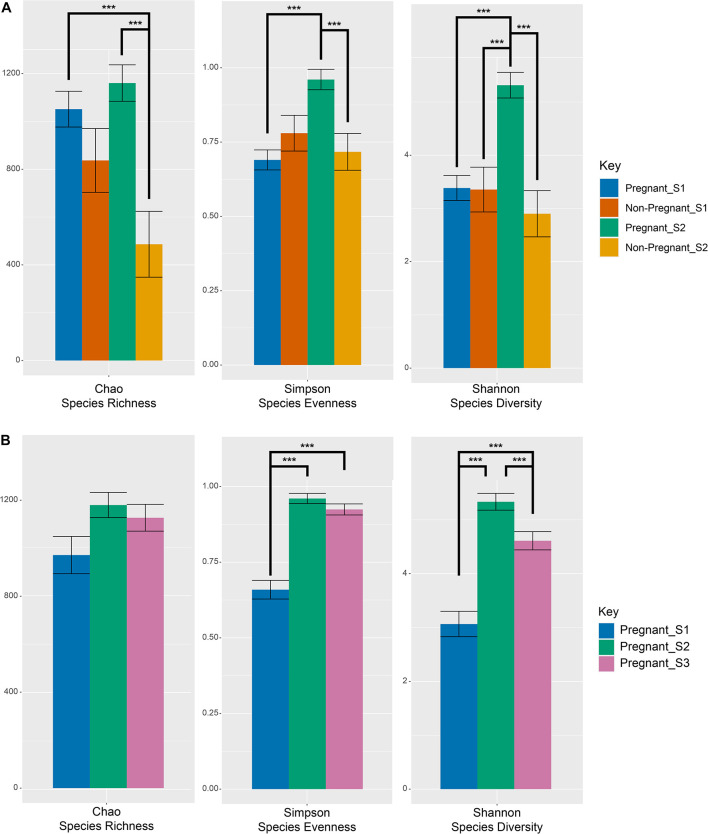
Measures of alpha-diversity indices of microbial OTUs at the S1 (pre-breeding) and S2 (after pregnancy testing) time points among pregnant and non-pregnant animals **(A)** as well as before and during gestation for successfully impregnated animals **(B)**. Error bars denote the standard error of the mean. Lines linking bars denote significant pairwise comparisons across the levels of the main effects. Main effects and interactions were tested using the models described in the statistics section (equation 1 and equation 2) and actual values can be found in Supplementary Tables 3, 8. Significant (*p* < 0.001) differences between specific pairwise comparisons are denoted with ***.

**FIGURE 2:**
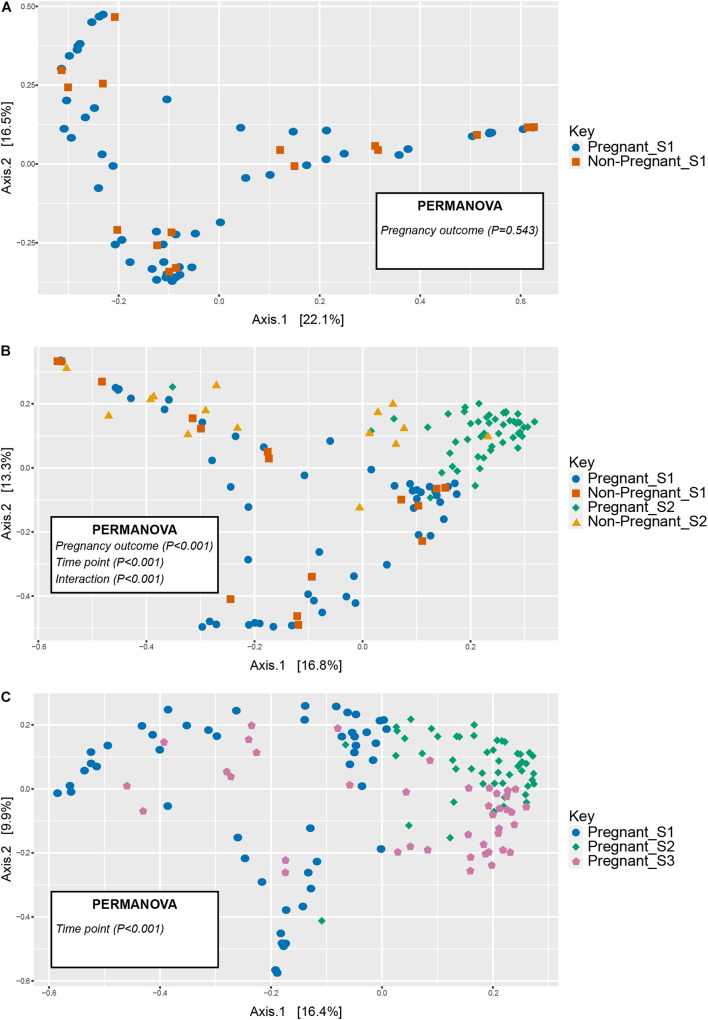
Unconstrained ordinations (principle coordinates analysis, PCoA) comparing vaginal microbial communities in animals that became pregnant or did not at **(A)** the pre-breeding time point (S1) and **(B)** over time (S1 and S2 time points), **(C)** as well as in pregnant animals before and during gestation (S1 through S3 time points). Distances between samples denote Bray-Curtis dissimilarity measures.

No differences were observed in microbial alpha-diversity (Shannon, *P* = 0.99), species richness (Chao, *P* = 0.5) and microbial community evenness (Simpson, *P* = 0.56) when comparing S1 samples obtained from ewes that were subsequently determined to be pregnant or non-pregnant (Figure 1A and Supplementary Table 3). Similarly, no differences in beta diversity were observed when comparing Bray-Curtis dissimilarities between these microbial communities. A visual representation of the Bray-Curtis dissimilarities between samples was made using an unconstrained ordination analysis (PCoA), which shows large overlap between these microbial communities at the pre-breeding time point (Figure 2A and Supplementary Tables 4, 5).

**FIGURE 3:**
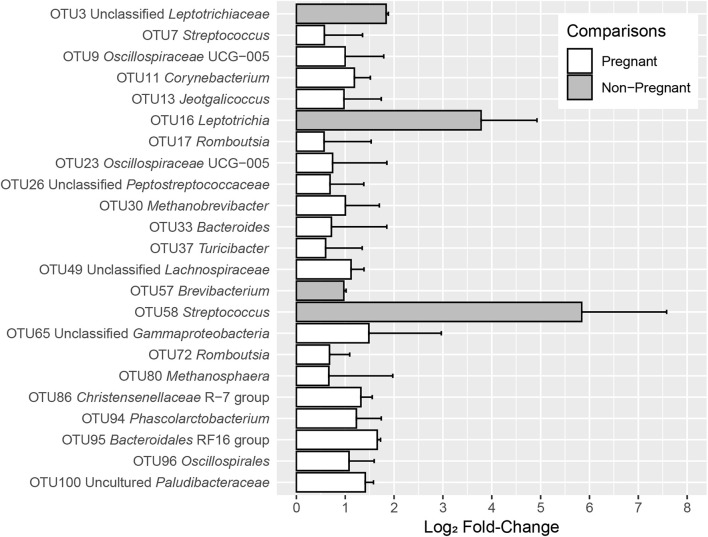
log2-fold changes of OTUs with significant differences between S1 and S2 sampling time points in pregnant and non-pregnant animals for the fixed effect of pregnancy outcome. The OTUs depicted in gray bars were more abundant in non-pregnant animals, whereas the OTUs depicted in white bars were more abundant in pregnant animals. Error bars indicate the absolute standard error of the mean.

Twenty-three OTUs differed (*q* < 0.05) among pregnant and non-pregnant ewes, irrespective of sampling time. Within the 100 most abundant OTUs, OTUs 3 (unclassified *Leptotrichiaceae*), 16 (*Leptotrichia*), 57 (*Brevibacterium*), and 58 (*Streptococcus*) were more abundant in non-pregnant animals at both time points, whereas nineteen OTUs were more abundant in pregnant animals (Figure 3 and Table 1). At the phylum level, *Fusobacteriota* was more abundant in non-pregnant animals, whereas *Euryarchaeota*, *Bacteroidota*, *Verrucomicrobiota*, *Desulfobacterota*, *Cyanobacteria*, *Fibrobacterota*, and *Deferribacterota* were more abundant in pregnant animals (Supplementary Table 6). OTUs that were significantly affected by the fixed effect of sampling time point can be found in Supplementary Figure 1.

**TABLE 1:** Differences in OTUs between vaginal microbiota of ewes with successful pregnancy outcome compared those with unsuccessful pregnancy outcome.

OTU	Taxonomy (Silva v138)	Comparison	More abundant in	Log2FC	*Q-*value
3	*Leptotrichiaceae* unclassified	Pregnant vs. Non-pregnant	Non-Pregnant	1.83	<0.001
7	*Streptococcus*	Pregnant vs. Non-pregnant	Pregnant	0.57	0.008
9	*Oscillospiraceae* UCG-005	Pregnant vs. Non-pregnant	Pregnant	0.99	<0.001
11	*Corynebacterium*	Pregnant vs. Non-pregnant	Pregnant	1.18	<0.001
13	*Jeotgalicoccus*	Pregnant vs. Non-pregnant	Pregnant	0.97	0.003
16	*Leptotrichia*	Pregnant vs. Non-pregnant	Non-Pregnant	3.78	0.003
17	*Romboutsia*	Pregnant vs. Non-pregnant	Pregnant	0.56	0.008
23	*Oscillospiraceae* UCG-005	Pregnant vs. Non-pregnant	Pregnant	0.74	0.006
26	*Peptostreptococcaceae* unclassified	Pregnant vs. Non-pregnant	Pregnant	0.68	0.004
30	*Methanobrevibacter*	Pregnant vs. Non-pregnant	Pregnant	1.0	<0.001
33	*Bacteroides*	Pregnant vs. Non-pregnant	Pregnant	0.71	0.011
37	*Turicibacter*	Pregnant vs. Non-pregnant	Pregnant	0.59	0.017
49	*Lachnospiraceae* unclassified	Pregnant vs. Non-pregnant	Pregnant	1.11	0.025
57	*Brevibacterium*	Pregnant vs. Non-pregnant	Non-Pregnant	0.97	0.005
58	*Streptococcus*	Pregnant vs. Non-pregnant	Non-Pregnant	5.84	<0.001
65	*Gammaproteobacteria* unclassified	Pregnant vs. Non-pregnant	Pregnant	1.48	0.029
72	*Romboutsia*	Pregnant vs. Non-pregnant	Pregnant	0.68	0.031
80	*Methanosphaera*	Pregnant vs. Non-pregnant	Pregnant	0.66	0.026
86	*Christensenellaceae* R-7 group	Pregnant vs. Non-pregnant	Pregnant	1.32	0.003
94	*Phascolarctobacterium*	Pregnant vs. Non-pregnant	Pregnant	1.23	0.002
95	*Bacteroidales* RF16 group	Pregnant vs. Non-pregnant	Pregnant	1.65	<0.001
96	*Oscillospirales*	Pregnant vs. Non-pregnant	Pregnant	1.08	0.005
100	*Paludibacteraceae* uncultured	Pregnant vs. Non-pregnant	Pregnant	1.41	0.007

*OTUs are significantly different based on fixed effect of pregnancy outcome with no interactions.*

**FIGURE 4:**
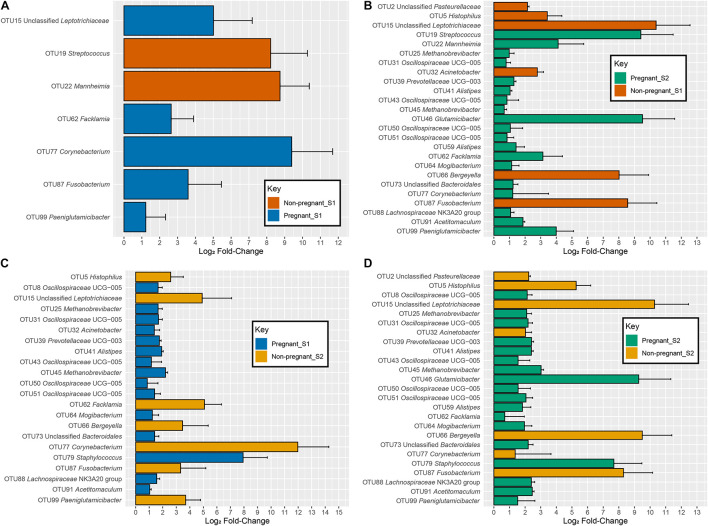
log2-fold changes of OTUs with a significant interaction between time point and pregnancy outcome specific to each comparison. **(A)** comparisons between non-pregnant-S1 (Orange) and pregnant-S1 (Blue), **(B)** comparisons between non-pregnant-S1 (Orange) and pregnant-S2 (Green), **(C)** comparisons between non-pregnant-S2 (Gold) and pregnant-S1 (Blue), and **(D)** comparisons between non-pregnant-S2 (Gold) and pregnant-S2 (Green). Error bars indicate the absolute standard error of the mean.

There was significant interaction between pregnancy outcome and sampling time point for seven OTUs (*q* < 0.05). At S1, OTUs 15 (*Leptotrichiaceae*), 62 (*Facklamia*), 77 (*Corynebacterium*), 87 (*Fusobacterium*), and 99 (*Paeniglutamicibacter*) were more abundant in animals that were not pregnant, whereas OTUs 19 (*Streptococcus*) and 22 (*Mannheimia*) were more abundant in animals that were pregnant (Figure 4A and Table 1).

### Comparison of Microbial Communities Over Time (S1 and S2) in Pregnant and Non-pregnant Ewes

**FIGURE 5:**
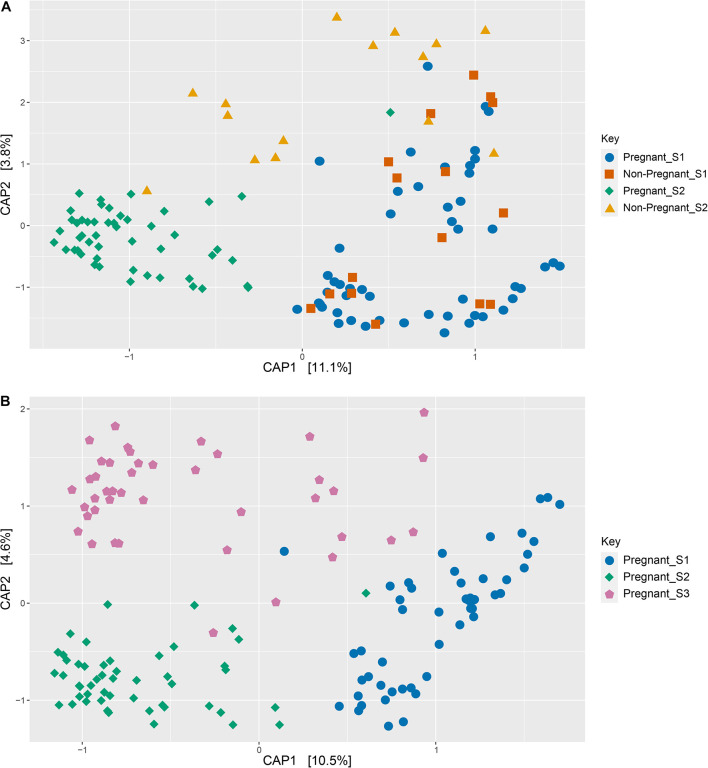
Constrained ordinations (canonical analysis of principle coordinates, CAP) comparing pregnant and non-pregnant vaginal microbial communities over time **(A)** as well as pregnant communities before and during gestation **(B)**. Distances between samples denote Bray-Curtis dissimilarity measures, and the data are constrained by variables of interest as defined in equations 1 and 2.

There was a significant interaction between pregnancy outcome and sampling time point for Chao, Simpson, and Shannon indices (Figure 1A and Supplementary Table 3, *P* < 0.05). Pregnant ewes at S2 had greater Chao, Simpson, and Shannon indices, relative to pregnant and non-pregnant animals at S1 or non-pregnant ewes at S2 (Figure 1A; *P* < 0.05). A distinct clustering of samples from pregnant animals at the S2 time point was observed in the unconstrained PCoA and the constrained CAP analyses (Figures 2B, 5A), and this response was confirmed by permutational multivariate analysis of the cluster variance (Pregnancy × Sampling, Supplementary Tables 4, 5, *P* < 0.05). The BetaDisperser test identified significant dispersion (variation) within these microbial communities for both fixed effects of pregnancy outcome and time point (Supplementary Table 4).

When considering OTUs with an interaction between pregnancy outcome and time point on abundance, several OTUs showed differences between the various groups. Fourteen OTUs −8 (*Oscillospiraceae* UCG-005), 25 (*Methanobrevibacter*), 31 (*Methanobrevibacter*), 39 (*Prevotellaceae* UCG-003), 41 (*Alistipes*), 43 (*Oscillospiraceae* UCG-005), 45 (*Methanobrevibacter*), 50 (*Oscillospiraceae* UCG-005), 51 (*Oscillospiraceae* UCG-005), 59 (*Alistipes*), 64 (*Mogibacterium*), 73 (*Bacteroidales*), 88 (*Lachnospiraceae*), and 90 (*Ruminococcaceae*) – increased in abundance in pregnant animals and decreased in abundance in non-pregnant animals from S1 to S2 (Supplementary Figure 2A and Figures 4B,C). The OTUs – 46 (*Glutamicibacter*), 62 (*Facklamia*), 77 (*Corynebacterium*), and 99 (*Paeniglutamicibacter*) – increased in both pregnant and non-pregnant animals from S1 to S2, but much larger increases were detected in animals that became pregnant. Conversely, OTUs 19 (*Streptococcus*) and 22 (*Mannheimia*) increased less drastically in pregnant animals than in non-pregnant animals (Supplementary Figure 2B, Figures 4B–D, and Supplementary Table 7).

The OTUs 2 (*Pasteurellaceae*), 5 (*Histophilus*), 15 (*Leptotrichiaceae*), 66 (*Bergeyella*), and 87 (*Fusobacterium*) decreased in abundance in pregnant animals and increased in abundance in non-pregnant animals from S1 to S2. Both pregnant and non-pregnant animals showed a decrease from S1 to S2 in OTUs 10 (*Staphylococcus*), 22 (*Mannheimia*), and 32 (*Acinetobacter*), but these OTUs were even less prevalent in non-pregnant animals. Conversely, OTU 79 (*Staphylococcus*) decreased more drastically in non-pregnant animals than in pregnant animals from S1 to S2. The log2-fold changes between the treatment groups and their associated taxonomy and values can be seen in Supplementary Table 7.

### Differences in Microbial Communities Before and During Gestation (S1, S2, and S3 Time Points)

Differences in ewe vaginal microbial community evenness were discovered when comparing the pre-breeding time point (S1) to the post-pregnancy check (S2) and late-pregnancy time points (S3) (Supplementary Table 8). Additionally, differences in species diversity were observed across all groups (Figure 1B). These differences in community structure were detectable using both an unconstrained ordination (PCoA) and constrained ordination (CAP) (Figures 2C, 5B). Similar to the previous S1 versus S2 comparison, community structure differences were further supported by differences in average community distances using PERMANOVA and in dispersion (variation) using BetaDisperser (Supplementary Table 9).

Several changes in OTU abundance were detected before and during gestation. Within the 100 most abundant OTUs, 63 OTUs significantly increased in pregnant animals at the S2 and S3 time points when compared to the S1 (pre-breeding) time point, whereas 16 OTUs decreased in pregnant ewes at both S2 and S3 time points when compared to the pre-breeding time point. The log2 fold-change between the significant OTUs can be viewed in Supplementary Figure 3, and their associated taxonomy and values can be found in Supplementary Table 10.

## Discussion

This study is among the first to characterize the ewe vaginal microbiota among pregnant and non-pregnant ewes (Serrano et al., 2020). Additionally, it is the first investigation to document ewe vaginal microbial communities over time during gestation. Results of our study lead us to a general consensus that the microbial communities within the ewe vagina are fundamentally different in animals with an established pregnancy when compared to their non-pregnant counterparts. At the pre-breeding time point (S1), microbial communities appeared to have high inter-individual variability in species and phylum abundances. During later time points in samples from pregnant animals, variability between samples decreased, leading to a more uniform average vaginal microbial community across samples. This was not the case for samples from animals that failed to establish and maintain a pregnancy, however, as they seemed to retain a non-uniform, fluctuating community structure.

The closest related strains or species and sequence similarities reported here rely on short (250 bp) PCR amplicons. It is widely recognized that classifications based on short 16S rRNA gene amplicons provide only limited taxonomic resolution. Thus, all similarities and closest related species reported in this manuscript represent approximations only. A more thorough comparison of the OTUs found here with other described species would require longer (full-length) 16S rRNA gene PCR amplicons. Furthermore, the paucity of 16S rRNA gene sequences from ruminant livestock (and particularly from sheep) vaginal microbial communities available for comparison also limits comparison of the sequences obtained here with other available sequencing data. Ewes from different breeds were sampled, and multiple mating sires were used during this study. Although the effects of breed and mating sire theoretically may contribute to microbial community composition in the reproductive tract, they were not the focal point of our study. Analyzing the ram’s reproductive tract microbiota by obtaining samples from the prepuce and/or from an ejaculate would be a relevant future research topic. Similarly, a more in-depth study of the effect of breed on reproductive tract microbiota is warranted.

A successful pregnancy may be difficult to predict based on the microbial communities that exist immediately prior to breeding. Although we attempted to identify potential biomarkers for successful establishment of pregnancy within the vaginal microbial communities present before breeding, we did not find an irrefutable marker. No differences in alpha diversity or Bray-Curtis dissimilarity (beta diversity) were detected at the pre-breeding time point in animals that did or did not become pregnant, suggesting that the overall community structure of successfully and unsuccessfully impregnated animals was highly similar on a whole-community level. Nevertheless, a small number of OTUs differed in abundance between animals that did and did not establish a successful pregnancy. The OTUs 15 (unclassified *Leptotrichaceae*), 62 (*Facklamia*), 77 (*Corynebacterium*), 87 (*Fusobacterium*), and 99 (*Paeniglutamicibacter*) were more abundant in animals that failed to establish a pregnancy, whereas OTUs 19 (*Streptococcus*) and 22 (*Mannheimia*) were more abundant in females that established and maintained a pregnancy.

The OTUs 15 and 87 were classified within the *Fusobacteriota* phylum. Members of the *Fusobacteriota* phylum are often found in the vaginal microbiota of ruminants, including sheep (Swartz et al., 2014; Serrano et al., 2020). Members within this phylum are known for contributing to bacterial vaginosis and pre-term births or stillbirths in humans, sheep and other ruminants (Galvão et al., 2019). *Fusobacteriota* are known to cause abortion in sheep (Boye et al., 2006), and they also contribute to the development of ovine foot rot (Zanolari et al., 2021). The OTU 77 was identified with 99.2% sequence similarity to *Corynebacterium glutamicum*, which is often considered non-pathogenic. Various *Corynebacterium* phylotypes and have been identified in the sheep vaginal microbiota in previous studies (Swartz et al., 2014; Serrano et al., 2020), as well as in cultivation-based studies (Sawyer, 1977; Manes et al., 2018). Certain members within the *Corynebacteriaceae* family are known ovine pathogens, such as *Corynebacterium pseudotuberculosis*, which causes caseous lymphadenitis (Gascoigne et al., 2020). However, it is currently unknown if these OTUs, which showed higher abundance in non-pregnant animals than in pregnant animals, contribute to the negative outcome of pregnancy. Future studies will be needed to investigate these phylotypes in more detail and their possible association with pregnancy outcome.

The OTU 19 was identified with 100% sequence similarity to *Streptococcus uberis*, which demonstrates gamma hemolysis and is known to cause mastitis in cows and sheep (Keane, 2019). Additionally, OTU 22 was identified as *Mannheimia haemolytica* (99.2% sequence similarity), which is known to cause mastitis and pneumonia in small ruminants (Omaleki et al., 2011; Besser et al., 2013; Confer and Ayalew, 2018; Katsafadou et al., 2019). It is unclear why these two phylotypes that are highly similar to pathogenic species are associated with the successful establishment of pregnancy. *Mannheimia*-like OTUs have been found in the vaginal microbiota of healthy dairy cattle and were more abundant in the vaginal microbiota of cows whose calves did not develop upper respiratory tract infections (Lima et al., 2019). The reproductive tract is not the site of pathogenesis for *Streptococcus uberis* or *Mannheimia haemolytica*, and although present, may not be negatively affecting the host’s reproductive function.

Samples were collected from all animals post-pregnancy testing (time point S2), which allowed us to include information about the vaginal microbiota over time in animals that failed to establish or maintain pregnancy (in the event of early embryonic death loss). Several differences in the microbiota were detected between pregnant and non-pregnant animals, likely driven by the establishment of pregnancy. Whole community species richness decreased in non-pregnant animals, and community evenness increased in pregnant animals at the S2 time point. These changes led to greater microbial community diversity in the pregnant animals at the S2 time point when compared to other time points. With this increase in diversity occurring only in pregnant animals at time point S2, we can infer that this change is occurring in tandem with pregnancy. This finding suggests that the vaginal microbial communities respond to the host physiological changes during gestation in a positive way, as more microbial diversity is often considered beneficial (Reese and Dunn, 2018), which aligns with successful pregnancy outcomes in this study.

The greatest physiological change associated with pregnancy is an increase in blood concentration of progesterone concomitant with the discontinuation of the waxing and waning of progesterone and estradiol-17β exhibited during the estrous cycle in non-pregnant animals. The elevated blood concentrations of progesterone observed during gestation lead to increased endometrial production of histotroph (to feed the preimplantation embryo) and increased viscosity of vagino-cervical mucus (to reduce likelihood of vaginal microbes entering the uterus and causing infection). Similar changes in microbial diversity have been observed in other ruminants as well, with a marked exception being humans where these trends are often reversed (Gupta et al., 2020).

The abundance of several OTUs increased in samples taken from pregnant ewes throughout the course of the pregnancy. Several of the OTUs that increased in abundance were classified within the relatively understudied *Oscillospiraceae* family. These organisms have never been cultured *in vitro* (Konikoff and Gophna, 2016), and very little is known about their metabolism. One recent study has described *Oscillospira*-like OTUs in the vaginal microbial communities of dairy cows (Lima et al., 2019). It has been suggested that, although often associated with fiber degradation and low body weights, *Oscillospira* may not degrade fiber but rather metabolize sugars released by host mucins, such as glucuronate (Konikoff and Gophna, 2016; Gophna et al., 2017). Using metagenome assembled genomes (MAGs) from the human gut, genes were found (Gophna et al., 2017) that shared homology to those coding for uronate isomerase, altronate oxidoreductases and altronate hydrolases, which are key steps in the breakdown of glucuronic acid. In addition to being a key component of heparin sulfate glycosaminoglycans, the glucuronate moiety is also a component in pregnanediol-3-glucuronide (PdG), a glucuronated form of progesterone (P4) that is excreted in the urine.

During the period of estrus in the ewe, ovulation occurs and the recently ruptured ovarian follicle is transformed into a corpus luteum (CL). The CL produces P4 which is important not only to stimulate endometrial glands to produce histotroph but also to inhibit myometrial (uterine muscle) contractions. Preventing uterine contractions is important to enable the conceptus to grow a placenta that subsequently attaches to the uterus to facilitate nutrient delivery to the developing fetus. Progesterone production by the ewe placenta eventually exceeds that of the CL and ensures maintenance of high concentrations of circulating P4. It is possible that *Oscillospira* are able to metabolize the P4, PdG or the glucuronate moiety appended to PdG that diffuses into the vaginal environment, leading to an increase of their overall abundance. The breakdown process of glucuronate to 2-dehydro-3-deoxy-D-gluconate releases two free hydrogen atoms, which may also help to explain the increase in methanogenic archaea (OTUs 25, 30, 45, and 80) found in the samples from pregnant ewes. A higher abundance of methanogenic archaea in pregnant animals has been described for vaginal microbial communities of Nellore cattle (Laguardia-Nascimento et al., 2015). Finally, several ORFs within the *Oscillospira* MAGs were identified as homologs to genes involved in butyrate production (Gophna et al., 2017). Butyrate is known to aid the host in forming tight cell junctions in the epithelial layer (Peng et al., 2009), potentially preventing bacterial infection that could ultimately lead to an abortion. Although future functional research is necessary, these microorganisms of the *Oscillospiraceae* family may be interacting mutualistically with the host and might have the potential to act as a biomarker for successful pregnancy.

Additionally, two OTUs (41 and 59) classified as *Alistipes* spp. increased in abundance in pregnant animals and decreased in non-pregnant animals from S1 to S2. Several species within the *Alistipes* genus have been described, and these species are correlated (both positively and negatively) with several diseases. *Alistipes* OTUs have previously been found in relatively high abundance in the vaginal microbiota of Nellore cattle (Laguardia-Nascimento et al., 2015). However, the function of *Alistipes* in the vaginal microbiota is currently unknown. It is thus unclear if the change in abundance of *Alistipes* is associated with pregnancy outcome.

Other OTUs increased in non-pregnant animals, such as OTU 2 (unclassified *Pasteurellaceae*), OTU 5 (*Histophilus*), OTU 15 (unclassified *Leptotrichiaceae*), OTU 66 (unclassified *Flavobacterium*), and OTU 87 (*Fusobacterium*). Species from both the *Fusobacterium* and *Histophilus* genera are known to cause reproductive disorders in sheep, including abortion in ewes (Boye et al., 2006) and epididymitis in rams (Sandal and Inzana, 2010; O’Toole and Sondgeroth, 2016). Individual *Histophilus* strains have been described as primary or opportunistic pathogens, or as commensals, but can also move between these classifications (Sandal and Inzana, 2010; O’Toole and Sondgeroth, 2016). In addition, some *Histophilus* isolates from the healthy genital tract often lack many of the known *Histophilus* virulence factors (Sandal and Inzana, 2010). Although no ewes aborted during this study, the presence of these bacteria may either be adversely affecting the establishment of pregnancy or having no adverse effects at all. Currently, a paucity of research exists tying *Leptotrichiaceae* and *Flavobacterium* to sheep health or performance (Leon-Vizcaino et al., 1987).

When comparing the microbiota from samples in ewes that became pregnant, the largest differences in microbial community structure were observed in the pre-breeding time point (S1) compared to S2 and S3. The S2 and S3 time points both showed higher microbial community evenness and diversity than S1. As noted previously, an even distribution of species is inversely correlated with dysbiosis - a disruption in these communities potentially resulting in loss of function. Additionally, the unconstrained ordination demonstrates the high amount of variation within the S1 samples, comparative to the other time points.

When comparing the S1 time point to the S2 time point, a high number of OTUs within the 100 most abundant OTUs showed significant changes in their abundance. A majority of these changes maintained the trend between S2 and S3 (increased or decreased compared to S1). Thirty-five of these OTUs maintained the trend and were differentially abundant based on the pregnancy outcome status discussed previously. Exploring the gene potential or expression from these consistent OTUs may help to identify microbial effects on establishing and maintaining pregnancy.

## Conclusion

We perform an in-depth characterization of the sheep vaginal microbiota and reveal distinct changes in microbial community diversity in relation to pregnancy and of microbial community composition before and during gestation. We also provide the first characterization of the sheep vaginal microbiota at multiple time points during gestation. Results of this study provide insights into potential biomarkers for pregnancy and point to OTUs which seem to correlate with ewe gestation. Further study of these OTUs may reveal new ways to address sheep reproductive efficiency. Future research may investigate the contribution of the mating sire’s reproductive tract microbiota on the ewe’s reproductive tract microbiota and possible implications toward pregnancy outcome. The potential effects of different dam and sire breeds and ages would also be relevant areas of future studies.

## Data Availability Statement

The datasets presented in this study can be found in online repositories. The names of the repository/repositories and accession number(s) can be found below: https://www.ncbi.nlm.nih.gov/, PRJNA739687.

## Ethics Statement

The animal study was reviewed and approved by the Iowa State University Institutional Animal Care and Use Committee (protocol no. 18-206).

## Author Contributions

SS-E and CY designed the study and obtained funding for it. CY and LK collected the samples. LK performed the experiments and analyzed the data. AP analyzed the sequencing data and performed statistical analysis of the sequencing data. LK, AP, CY, and SS-E wrote the manuscript. All authors reviewed and approved the final manuscript.

## Conflict of Interest

The authors declare that the research was conducted in the absence of any commercial or financial relationships that could be construed as a potential conflict of interest.

## Publisher’s Note

All claims expressed in this article are solely those of the authors and do not necessarily represent those of their affiliated organizations, or those of the publisher, the editors and the reviewers. Any product that may be evaluated in this article, or claim that may be made by its manufacturer, is not guaranteed or endorsed by the publisher.
