# Supplementary figures and images for: Ewe Vaginal Microbiota: Associations With Pregnancy Outcome and Changes During Gestation
Source: Front Microbiol. 2021 Oct 22;12:745884. doi: 10.3389/fmicb.2021.745884 (PMC8570082; doi:10.3389/fmicb.2021.745884)

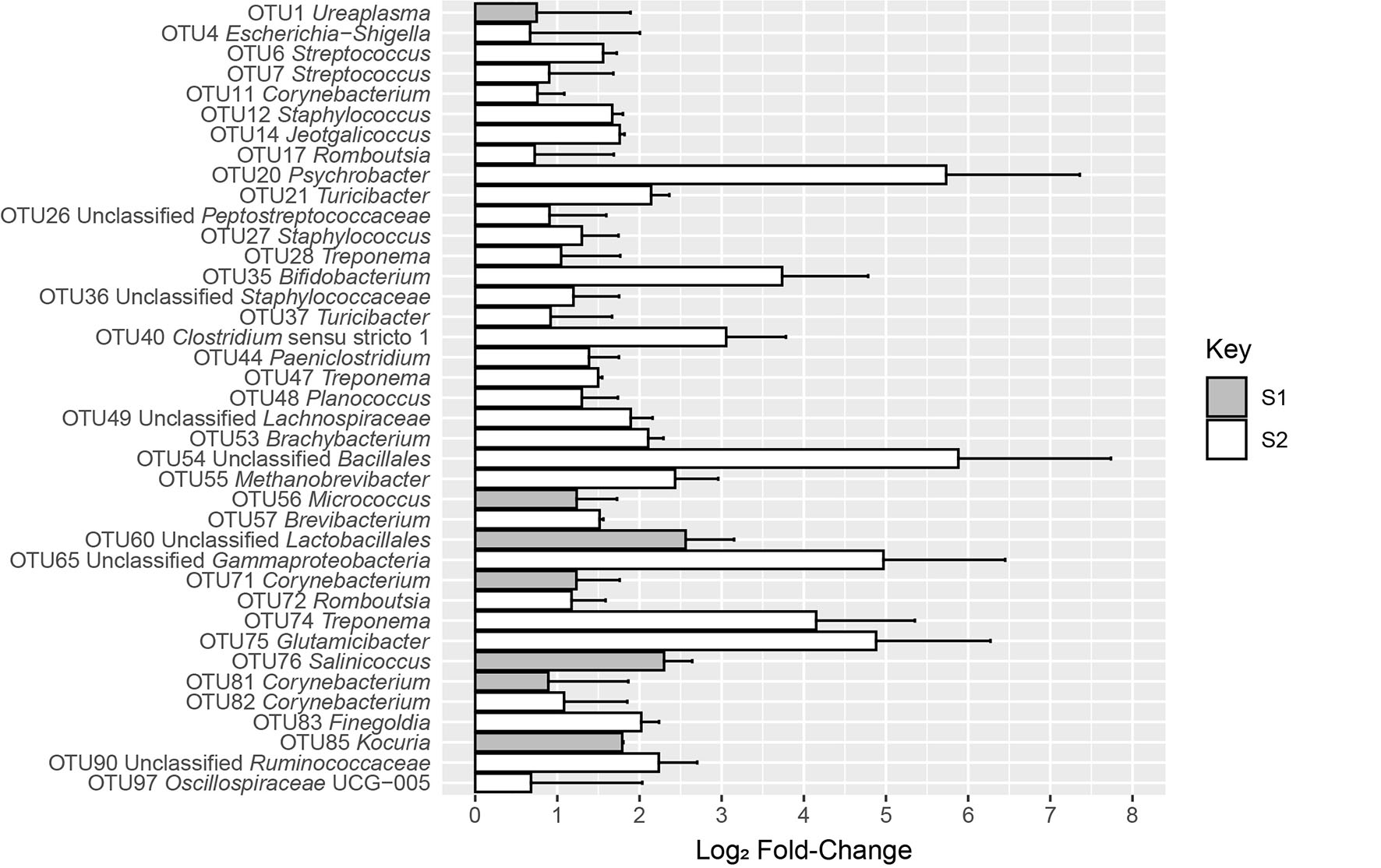

Supplement: Supplementary file 1 [file Image_1.JPEG]

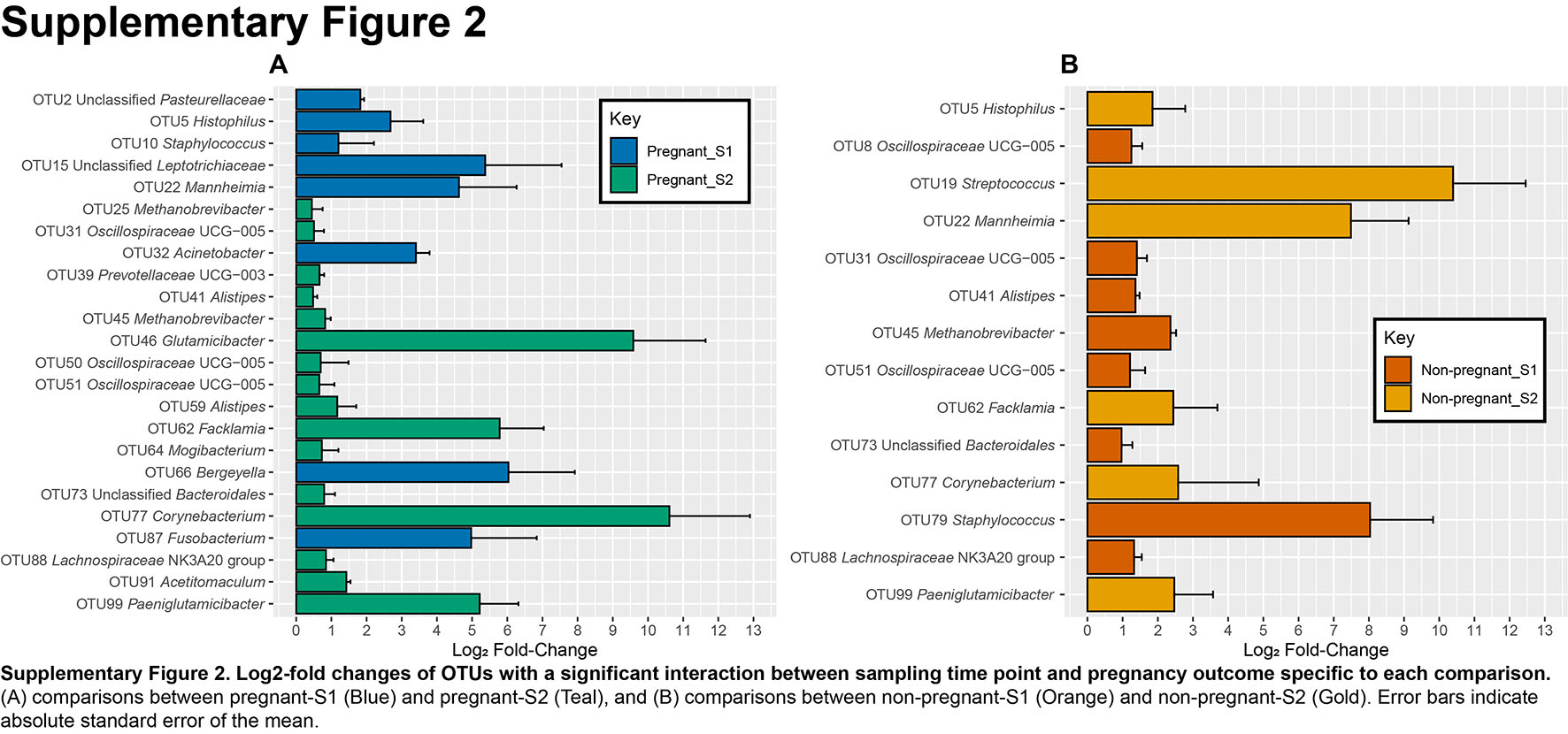

Supplement: Supplementary file 2 [file Image_2.JPEG]

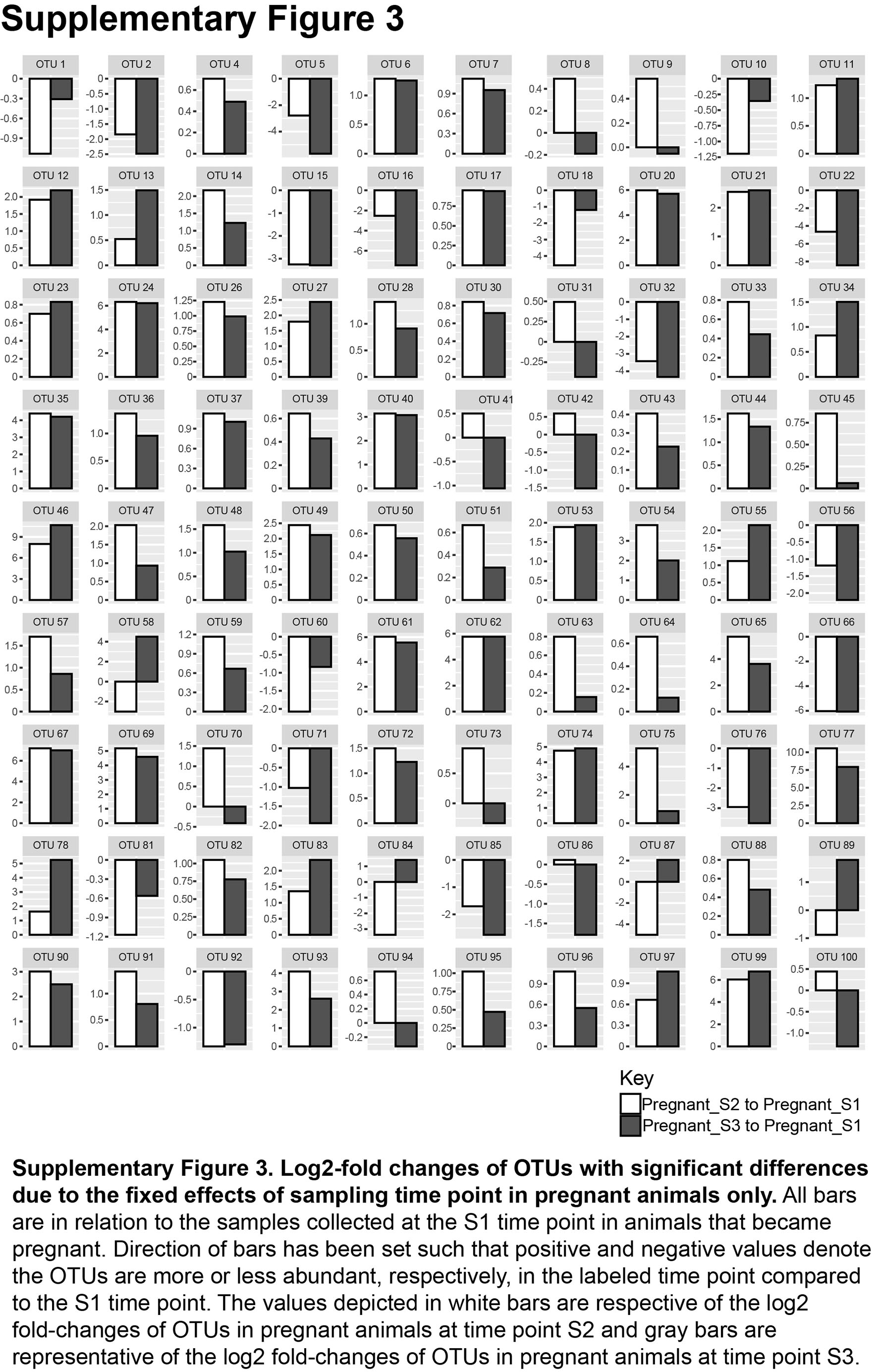

Supplement: Supplementary file 3 [file Image_3.JPEG]
