# Supplementary material for: Ewe Vaginal Microbiota: Associations With Pregnancy Outcome and Changes During Gestation
Source: Front Microbiol. 2021 Oct 22;12:745884. doi: 10.3389/fmicb.2021.745884 (PMC8570082; doi:10.3389/fmicb.2021.745884)
Supplement: Supplementary file 4 [file Table_1.PDF]

Supplementary Table S1. Relative abundance of bacterial phyla in all vaginal samples from ewes used in this study.

Phyla	Relative abundance (%)
Firmicutes	52.99
Proteobacteria	16.28
Bacteroidota	11.33
Actinobacteriota	6.70
Fusobacteriota	6.28
Spirochaetota	1.81
Euryarchaeota	1.74
Campilobacterota	0.81
Verrucomicrobiota	0.78
Cyanobacteria	0.32
Desulfobacterota	0.22
Fibrobacterota	0.21
Chloroflexi	0.10
Planctomycetota	0.09
Acidobacteriota	0.06
Elusimicrobiota	0.04
Crenarchaeota	0.04
Thermoplasmatota	0.03
Halobacterota	0.03
Deinococcota	0.02
Synergistota	0.02
Patescibacteria	0.02
Gemmatimonadota	0.02
Bdellovibrionota	0.01
Sumerlaeota	0.01
Armatimonadota	0.01
Deferribacterota	0.01
Abditibacteriota	0.01
Halanaerobiaeota	<0.01
Latescibacterota	<0.01
Nitrospirota	<0.01
Dadabacteria	<0.01

Supplementary Table S2. The 50 most abundant OTUs across all vaginal samples from ewes used in this study.

OTU	Relative abundance (%)	Phylum	Taxonomy (Silva v138)	NCBI BLAST Classification	Similarity (%)	Accession no.
OTU 1	9.94	*Firmicutes*	*Ureaplasma*	*Ureaplasma diversum*	97.6	MW090794.1
OTU 2	4.95	*Proteobacteria*	Unc. *Pasteurellaceae*	*Actinobacillus seminis*	99.6	NR_042872.1
OTU 3	4.03	*Fusobacteriota*	Unc. *Leptotrichiaceae*	*Streptobacillus canis*	96.8	MN636430.1
OTU 4	2.67	*Proteobacteria*	*Escherichia-Shigella*	*Shigella flexneri*	99.6	JQ680820.1
OTU 5	2.55	*Proteobacteria*	*Histophilus*	*Histophilus somni*	99.6	CP042983.1
OTU 6	2.13	*Firmicutes*	*Streptococcus*	*Streptococcus pluranimalium*	100	LC316932.1
OTU 7	1.49	*Firmicutes*	*Streptococcus*	*Streptococcus lutetiensis*	100	MT626096.1
OTU 8	1.16	*Firmicutes*	*Oscillospiraceae* UCG-005	*Pseudoflavonifractor sp. strain MSJ-30*	96.8	MZ310616.1
OTU 9	1.06	*Firmicutes*	*Oscillospiraceae* UCG-005	*Pseudoflavonifractor sp. strain MSJ-30*	98.4	MZ310616.1
OTU 10	0.96	*Firmicutes*	*Staphylococcus*	*Staphylococcus pseudintermedius*	99.6	MT510359.1
OTU 11	0.94	*Actinobacteriota*	*Corynebacterium*	*Corynebacterium glutamicum*	99.2	AP022856.1
OTU 12	0.90	*Firmicutes*	*Staphylococcus*	*Staphylococcus equorum*	99.6	MN918391.1
OTU 13	0.83	*Firmicutes*	*Jeotgalicoccus*	*Jeotgalicoccus psychorphilus*	99.6	MT793509.1
OTU 14	0.75	*Firmicutes*	*Jeotgalicoccus*	*Jeotgalicoccus coquinae*	99.6	NR_117490.1
OTU 15	0.73	*Fusobacteriota*	Unc. *Leptotrichiaceae*	*Oceanivirga miroungae strain ES3154-GLU*	94.1	MK828132.1
OTU 16	0.69	*Fusobacteriota*	*Leptotrichia*	*Leptotrichia goodfellowii*	95.7	MT242540.1
OTU 17	0.67	*Firmicutes*	*Romboutsia*	*Romboutsia timonensis*	99.6	NR_144740.1
OTU 18	0.67	*Firmicutes*	Unc. *Aerococcaceae*	*Aerococcus urinaehominis*	99.6	CP014163.1
OTU 19	0.65	*Firmicutes*	*Streptococcus*	*Streptococcus uberis*	100	LC316933.1
OTU 20	0.61	*Proteobacteria*	*Psychrobacter*	*Psychrobacter pulmonis*	99.6	MT225754.1
OTU 21	0.56	*Firmicutes*	*Turicibacter*	*Turicibacter sanguinis*	99.6	LC515561.1
OTU 22	0.51	*Proteobacteria*	*Mannheimia*	*Mannheimia haemolytica*	99.2	MN907437.1
OTU 23	0.51	*Firmicutes*	*Oscillospiraceae* UCG-005	*Oscillibacter sp. strain 128x*	94.49	MK287653.1
OTU 24	0.47	*Proteobacteria*	*Psychrobacter*	*Psychrobacter submarinus*	99.6	MK123477.1
OTU 25	0.46	*Euryarchaeota*	*Methanobrevibacter*	*Methanobrevibacter millerae*	99.6	CP011266.1

OTU 26	0.44	*Firmicutes*	Unc. *Peptostreptococcaceae*	*Clostridioides difficile*	99.2	CP035499.1
OTU 27	0.44	*Firmicutes*	*Staphylococcus*	*Staphylococcus epidermidis*	99.6	MZ255839.1
OTU 28	0.40	*Spirochaetota*	*Treponema*	*Treponema brennaborense*	91.3	NR_029348.1
OTU 29	0.40	*Proteobacteria*	*Mannheimia*	*Mannheimia haemolytica*	100	CP023044.1
OTU 30	0.39	*Euryarchaeota*	*Methanobrevibacter*	*Methanobrevibacter ruminantium*	99.6	KP123415.1
OTU 31	0.39	*Firmicutes*	*Oscillospiraceae* UCG-005	*Pseudoflavonifractor sp. strain MSJ-30*	96.8	MZ310616.1
OTU 32	0.38	*Proteobacteria*	*Acinetobacter*	*Acinetobacter variabilis*	99.6	MN932362.1
OTU 33	0.38	*Bacteroidota*	*Bacteroides*	*Bacteroides plebeius*	92.9	MT749279.1
OTU 34	0.37	*Firmicutes*	*Anaerococcus*	*Anaerococcus nagyae*	94.8	NR_146835.1
OTU 35	0.36	*Actinobacteriota*	*Bifidobacterium*	*Bifidobacterium pseudolongum*	99.6	LC527458.1
OTU 36	0.34	*Firmicutes*	Unc. *Staphylococcaceae*	*Salinicoccus alkaliphilus*	99.6	MH586685.1
OTU 37	0.32	*Firmicutes*	*Turicibacter*	*Turicibacter sanguinis*	99.2	LC515561.1
OTU 38	0.31	*Firmicutes*	*Staphylococcus*	*Staphylococcus aureus*	99.6	MT628394.1
OTU 39	0.31	*Bacteroidota*	*Prevotellaceae* UCG-003	*Paraprevotella clara*	87.7	NR_113077.1
OTU 40	0.30	*Firmicutes*	*Clostridium_sensu_stricto_1*	*Clostridium saudiense*	100	LC515546.1
OTU 41	0.29	*Bacteroidota*	*Alistipes*	*Alistipes finegoldii*	96.1	NR_102944.1
OTU 42	0.27	*Firmicutes*	Unc. *Planococcaceae*	*Lysinibacillus halotolerans*	99.6	MT487702.1
OTU 43	0.26	*Firmicutes*	*Oscillospiraceae* UCG-005	*Pseudoflavonifractor sp. strain MSJ-30*	96.4	MZ310616.1
OTU 44	0.26	*Firmicutes*	*Paeniclostridium*	*Eubacterium tenue*	99.6	LC011042.1
OTU 45	0.26	*Euryarchaeota*	*Methanobrevibacter*	*Methanobrevibacter thaueri*	98.8	NR_044787.1
OTU 46	0.25	*Actinobacteriota*	*Glutamicibacter*	*Glutamicibacter bergerei*	99.6	MT534553.1
OTU 47	0.25	*Spirochaetota*	*Treponema*	*Treponema succinifaciens*	88.93	NR_074755.1
OTU 48	0.23	*Firmicutes*	*Planococcus*	*Planomicrobium flavidum*	99.6	MN931359.1
OTU 49	0.23	*Firmicutes*	Unc. *Lachnospiraceae*	*Lactonifactor longoviformis*	96.4	LT223592.1
OTU 50	0.23	*Firmicutes*	*Oscillospiraceae* UCG-005	*Pseudoflavonifractor sp. strain MSJ-30*	95.7	MZ310616.1

Supplementary Table S3. Alpha diversity comparison results when using Eq. 1 to determine differences in vaginal microbiota between pregnant and non-pregnant animals over time (S1 and S2). Including significant pairwise comparisons.

Chao Species Richness

Type 3 Tests of Fixed Effects

Effect	Num DF	Den DF	F Value	Pr > F
sampling	1	60.4	1.37	0.25
bred	1	60.7	14.71	0.0003
sampling*bred	1	60.4	4.95	0.03

Significant pairwise comparisons of Chao Species Richness estimates values across levels of fixed effects

Effect	Pairs			Estimate	SE	DF	t value	P values	Adjustment	Q value
sampling*bred	Pregnant_S1	vs	Non-pregnant_S2	565	156.72	125	3.61	<0.001	Tukey-Kramer	0.0035
sampling*bred	Pregnant_S2	vs	Non-pregnant_S2	674.01	157.44	126	4.28	<0.001	Tukey-Kramer	<0.001

Simpson Evenness

Type 3 Tests of Fixed Effects

Effect	Num DF	Den DF	F Value	Pr > F
sampling	1	63.8	4.44	0.04
bred	1	63.9	2.38	0.13
sampling*bred	1	63.8	11.44	0.001

Significant pairwise comparisons of Simpson Evenness estimates values across levels of fixed effects

Effect	Pairs			Estimate	SE	DF	t value	P value	Adjustment	Q value
sampling*bred	Pregnant_S1	vs	Pregnant_S2	-0.270	0.048	63.2	-5.64	<0.001	Tukey-Kramer	<0.001
sampling*bred	Pregnant_S2	vs	Non-pregnant_S2	0.243	0.071	127	3.43	<0.001	Tukey-Kramer	0.006

Shannon Diversity

Type 3 Tests of Fixed Effects				
Effect	Num DF	Den DF	F Value	Pr > F
sampling	1	61.9	5.22	0.0257
bred	1	62.3	11.08	0.0015
sampling*bred	1	61.9	13.63	0.0005

Significant pairwise comparisons of Shannon Diversity estimates values across levels of fixed effects

Effect	Pairs			Estimate	SE	DF	t Value	P values	Adjustment	Q value
sampling*bred	Pregnant_S1	vs	Pregnant_S2	-1.932	0.314	61.3	-6.15	<0.001	Tukey-Kramer	<0.001
sampling*bred	Non-pregnant_S1	vs	Pregnant_S2	-1.961	0.485	125	-4.05	<0.001	Tukey-Kramer	<0.001
sampling*bred	Pregnant_S2	vs	Non-pregnant_S2	2.416	0.496	125	4.87	<0.001	Tukey-Kramer	<0.001

Supplementary Table S4. PERMANOVA and BetaDisperser results when using Eq. 1 to determine differences in vaginal microbiota between pregnant and non-pregnant animals over time (S1 and S2).

PERMANOVA

adonis(formula = data_bray ~ sampling + Pregnancy_Outcome + sampling * Pregnancy_Outcome, data = data_sub_type_df)

Permutation: free

Number of permutations: 999

	Df	SumsOfSqs	MeanSqs	F.Model	R2	Pr(>F)
sampling	1	4.572	4.5718	14.273	0.09525	0.001
Pregnancy_Outcome	1	1.669	1.6692	5.2113	0.03478	0.001
sampling: Pregnancy_Outcome	1	1.078	1.078	3.3653	0.02246	0.001
Residuals	127	40.68	0.3203		0.84752	
Total	130	47.999			1	

Betadisperser

Sampling

Permutation test for homogeneity of multivariate dispersions

Permutation: free

Number of permutations: 999

Response: Distances

	Df	Sum Sq	Mean Sq	F	N.Perm	Pr(>F)
Groups	1	0.16147	0.16147	10.448	999	0.002
Residuals	130	2.00913	0.015455			

Pregnancy Outcome

Permutation test for homogeneity of multivariate dispersions

Permutation: free

Number of permutations: 999

Response: Distances

	Df	Sum Sq	Mean Sq	F	N.Perm	Pr(>F)
Groups	1	0.06042	0.060422	6.5184	999	0.011
Residuals	130	1.20503	0.009269			

Supplementary Table S5. Pairwise PERMANOVA results when using Eq. 1 comparing different levels of time point and pregnancy status differences in vaginal microbiota between pregnant and non-pregnant animals over time (S1 and S2).

Pairwise PERMANOVA

	pairs		Df	SumsOfSqs	F.Model	R2	p.value	p.adjusted	sig
Pregnant_S2	vs	Pregnant_S1	1	4.692134	15.67839	0.137919	0.001	0.006	**
Pregnant_S2	vs	Non-pregnant_S2	1	2.4347759	9.215103	0.129398	0.001	0.006	**
Pregnant_S2	vs	Non-pregnant_S1	1	2.4073361	8.761657	0.122094	0.001	0.006	**
Pregnant_S1	vs	Non-pregnant_S2	1	1.9322458	5.320589	0.076753	0.001	0.006	**
Pregnant_S1	vs	Non-pregnant_S1	1	0.3255337	0.875421	0.013289	0.549	1	
Non-pregnant_S2	vs	Non-pregnant_S1	1	0.9516895	2.459059	0.078167	0.005	0.03	*

Supplementary Table S6. Differences in phyla between vaginal microbiota of ewes with successful pregnancy outcome compared those with unsuccessful pregnancy outcome at both the S1 and S2 time point. Phyla are significantly different based on fixed effect of pregnancy outcome with no interactions between the fixed effects.

**Taxonomy (Silva v138)**	**Comparison**	**More abundant in**	**Log2FC**	***Q*- value**
Fusobacteriota	Pregnant vs. Non-pregnant	Non-Pregnant	1.92	<0.001
Euryarchaeota	Pregnant vs. Non-pregnant	Pregnant	0.56	0.023
Bacteroidota	Pregnant vs. Non-pregnant	Pregnant	0.33	0.002
Verrucomicrobiota	Pregnant vs. Non-pregnant	Pregnant	0.71	0.005
Desulfobacterota	Pregnant vs. Non-pregnant	Pregnant	0.60	0.025
Cyanobacteria	Pregnant vs. Non-pregnant	Pregnant	0.55	0.011
Fibrobacterota	Pregnant vs. Non-pregnant	Pregnant	0.95	0.024
Deferribacterota	Pregnant vs. Non-pregnant	Pregnant	3.17	0.044
Sumerlaeota	Pregnant vs. Non-pregnant	Pregnant	2.79	0.001

Supplementary Table 7. Differences in OTUs between vaginal microbiota of ewes with successful pregnancy outcome compared those with unsuccessful pregnancy outcome over time (S1, S2). OTUs are significantly different based on interaction effect of pregnancy outcome and time point.

OTU	Taxonomy (Silva v138)	Comparison	More abundant in	Log2FC	Q- value
OTU 2	unclassified *Pasteurellaceae*	Pregnant-S2 vs Non-Pregnant-S1	Non-Pregnant-S1	2.15	0.015
OTU 2	unclassified *Pasteurellaceae*	Pregnant-S2 vs Non-Pregnant-S2	Non-Pregnant-S2	2.23	0.015
OTU 5	*Histophilus*	Pregnant-S2 vs Non-Pregnant-S1	Non-Pregnant-S1	3.42	0.002
OTU 5	*Histophilus*	Pregnant-S1 vs Non-Pregnant-S2	Non-Pregnant-S2	2.59	0.002
OTU 5	*Histophilus*	Pregnant-S2 vs Non-Pregnant-S2	Non-Pregnant-S2	5.27	0.002
OTU 8	*Oscillospiraceae* UCG-005	Pregnant-S1 vs Non-Pregnant-S2	Pregnant-S1	1.66	0.031
OTU 8	*Oscillospiraceae* UCG-005	Pregnant-S2 vs Non-Pregnant-S2	Pregnant-S2	2.14	0.031
OTU 15	unclassified *Leptotrichiaceae*	Pregnant-S1 vs Non-Pregnant-S1	Non-Pregnant-S1	5.01	0.048
OTU 15	unclassified *Leptotrichiaceae*	Pregnant-S2 vs Non-Pregnant-S1	Non-Pregnant-S1	10.38	0.048
OTU 15	unclassified *Leptotrichiaceae*	Pregnant-S1 vs Non-Pregnant-S2	Non-Pregnant-S2	4.91	0.048
OTU 15	unclassified *Leptotrichiaceae*	Pregnant-S2 vs Non-Pregnant-S2	Non-Pregnant-S2	10.28	0.048
OTU 19	*Streptococcus*	Pregnant-S1 vs Non-Pregnant-S1	Pregnant-S1	8.21	<0.001
OTU 19	*Streptococcus*	Pregnant-S2 vs Non-Pregnant-S1	Pregnant-S2	9.41	<0.001
OTU 22	*Mannheimia*	Pregnant-S1 vs Non-Pregnant-S1	Pregnant-S1	8.74	<0.001
OTU 22	*Mannheimia*	Pregnant-S2 vs Non-Pregnant-S1	Pregnant-S2	4.11	<0.001
OTU 25	*Methanobrevibacter*	Pregnant-S2 vs Non-Pregnant-S1	Pregnant-S2	0.99	0.048
OTU 25	*Methanobrevibacter*	Pregnant-S1 vs Non-Pregnant-S2	Pregnant-S1	1.66	0.048
OTU 25	*Methanobrevibacter*	Pregnant-S2 vs Non-Pregnant-S2	Pregnant-S2	2.10	0.048
OTU 31	*Oscillospiraceae* UCG-005	Pregnant-S2 vs Non-Pregnant-S1	Pregnant-S2	0.79	0.002
OTU 31	*Oscillospiraceae* UCG-005	Pregnant-S1 vs Non-Pregnant-S2	Pregnant-S1	1.69	0.002
OTU 31	*Oscillospiraceae* UCG-005	Pregnant-S2 vs Non-Pregnant-S2	Pregnant-S2	2.19	0.002
OTU 32	*Acinetobacter*	Pregnant-S2 vs Non-Pregnant-S1	Non-Pregnant-S1	2.78	0.024
OTU 32	*Acinetobacter*	Pregnant-S1 vs Non-Pregnant-S2	Pregnant-S1	1.38	0.024
OTU 32	*Acinetobacter*	Pregnant-S2 vs Non-Pregnant-S2	Non-Pregnant-S2	2.02	0.024

OTU 39	*Prevotellaceae* UCG-003	Pregnant-S2 vs Non-Pregnant-S1	Pregnant-S2	1.28	0.027
OTU 39	*Prevotellaceae* UCG-003	Pregnant-S1 vs Non-Pregnant-S2	Pregnant-S1	1.75	0.027
OTU 39	*Prevotellaceae* UCG-003	Pregnant-S2 vs Non-Pregnant-S2	Pregnant-S2	2.41	0.027
OTU 41	*Alistipes*	Pregnant-S2 vs Non-Pregnant-S1	Pregnant-S2	1.04	0.008
OTU 41	*Alistipes*	Pregnant-S1 vs Non-Pregnant-S2	Pregnant-S1	1.92	0.008
OTU 41	*Alistipes*	Pregnant-S2 vs Non-Pregnant-S2	Pregnant-S2	2.40	0.008
OTU 43	*Oscillospiraceae* UCG-005	Pregnant-S2 vs Non-Pregnant-S1	Pregnant-S2	0.84	0.031
OTU 43	*Oscillospiraceae* UCG-005	Pregnant-S1 vs Non-Pregnant-S2	Pregnant-S1	1.16	0.031
OTU 43	*Oscillospiraceae* UCG-005	Pregnant-S2 vs Non-Pregnant-S2	Pregnant-S2	1.55	0.031
OTU 45	*Methanobrevibacter*	Pregnant-S2 vs Non-Pregnant-S1	Pregnant-S2	0.66	0.001
OTU 45	*Methanobrevibacter*	Pregnant-S1 vs Non-Pregnant-S2	Pregnant-S1	2.20	0.001
OTU 45	*Methanobrevibacter*	Pregnant-S2 vs Non-Pregnant-S2	Pregnant-S2	3.02	0.001
OTU 46	*Glutamicibacter*	Pregnant-S2 vs Non-Pregnant-S1	Pregnant-S2	9.52	<0.001
OTU 46	*Glutamicibacter*	Pregnant-S2 vs Non-Pregnant-S2	Pregnant-S2	9.27	<0.001
OTU 50	*Oscillospiraceae* UCG-005	Pregnant-S2 vs Non-Pregnant-S1	Pregnant-S2	1.06	0.049
OTU 50	*Oscillospiraceae* UCG-005	Pregnant-S1 vs Non-Pregnant-S2	Pregnant-S1	0.86	0.002
OTU 50	*Oscillospiraceae* UCG-005	Pregnant-S2 vs Non-Pregnant-S2	Pregnant-S2	1.55	0.049
OTU 51	*Oscillospiraceae* UCG-005	Pregnant-S2 vs Non-Pregnant-S1	Pregnant-S2	0.85	0.005
OTU 51	*Oscillospiraceae* UCG-005	Pregnant-S1 vs Non-Pregnant-S2	Pregnant-S1	1.40	0.024
OTU 51	*Oscillospiraceae* UCG-005	Pregnant-S2 vs Non-Pregnant-S2	Pregnant-S2	2.06	0.005
OTU 59	*Alistipes*	Pregnant-S2 vs Non-Pregnant-S1	Pregnant-S2	1.42	0.031
OTU 59	*Alistipes*	Pregnant-S2 vs Non-Pregnant-S2	Pregnant-S2	1.83	0.031
OTU 62	*Facklamia*	Pregnant-S1 vs Non-Pregnant-S1	Non-Pregnant-S1	2.64	<0.001
OTU 62	*Facklamia*	Pregnant-S2 vs Non-Pregnant-S1	Pregnant-S2	3.14	<0.001
OTU 62	*Facklamia*	Pregnant-S1 vs Non-Pregnant-S2	Non-Pregnant-S2	5.09	<0.001
OTU 62	*Facklamia*	Pregnant-S2 vs Non-Pregnant-S2	Pregnant-S2	0.70	<0.001
OTU 64	*Mogibacterium*	Pregnant-S2 vs Non-Pregnant-S1	Pregnant-S2	1.14	0.027

OTU 64	*Mogibacterium*	Pregnant-S1 vs Non-Pregnant-S2	Pregnant-S1	1.23	0.027
OTU 64	*Mogibacterium*	Pregnant-S2 vs Non-Pregnant-S2	Pregnant-S2	1.96	0.027
OTU 66	*Bergeyella*	Pregnant-S2 vs Non-Pregnant-S1	Non-Pregnant-S1	8.02	0.001
OTU 66	*Bergeyella*	Pregnant-S1 vs Non-Pregnant-S2	Non-Pregnant-S2	3.47	0.001
OTU 66	*Bergeyella*	Pregnant-S2 vs Non-Pregnant-S2	Non-Pregnant-S2	9.50	0.001
OTU 73	unclassified *Bacteroidales*	Pregnant-S2 vs Non-Pregnant-S1	Pregnant-S2	1.23	0.003
OTU 73	unclassified *Bacteroidales*	Pregnant-S1 vs Non-Pregnant-S2	Pregnant-S1	1.40	0.003
OTU 73	unclassified *Bacteroidales*	Pregnant-S2 vs Non-Pregnant-S2	Pregnant-S2	2.20	0.003
OTU 77	*Corynebacterium*	Pregnant-S1 vs Non-Pregnant-S1	Non-Pregnant-S1	9.40	<0.001
OTU 77	*Corynebacterium*	Pregnant-S2 vs Non-Pregnant-S1	Pregnant-S2	1.21	<0.001
OTU 77	*Corynebacterium*	Pregnant-S1 vs Non-Pregnant-S2	Non-Pregnant-S2	11.97	<0.001
OTU 77	*Corynebacterium*	Pregnant-S2 vs Non-Pregnant-S2	Non-Pregnant-S2	1.37	<0.001
OTU 79	*Staphylococcus*	Pregnant-S1 vs Non-Pregnant-S2	Pregnant-S1	7.93	0.004
OTU 79	*Staphylococcus*	Pregnant-S2 vs Non-Pregnant-S2	Pregnant-S2	7.68	0.004
OTU 87	*Fusobacterium*	Pregnant-S1 vs Non-Pregnant-S1	Non-Pregnant-S1	3.59	0.034
OTU 87	*Fusobacterium*	Pregnant-S2 vs Non-Pregnant-S1	Non-Pregnant-S1	8.57	0.034
OTU 87	*Fusobacterium*	Pregnant-S1 vs Non-Pregnant-S2	Non-Pregnant-S2	3.32	0.034
OTU 87	*Fusobacterium*	Pregnant-S2 vs Non-Pregnant-S2	Non-Pregnant-S2	8.30	0.034
OTU 88	*Lachnospiraceae* NK3A20 group	Pregnant-S2 vs Non-Pregnant-S1	Pregnant-S2	1.07	0.003
OTU 88	*Lachnospiraceae* NK3A20 group	Pregnant-S1 vs Non-Pregnant-S2	Pregnant-S1	1.56	0.003
OTU 88	*Lachnospiraceae* NK3A20 group	Pregnant-S2 vs Non-Pregnant-S2	Pregnant-S2	2.40	0.003
OTU 91	*Acetitomaculum*	Pregnant-S2 vs Non-Pregnant-S1	Pregnant-S2	1.87	0.004
OTU 91	*Acetitomaculum*	Pregnant-S1 vs Non-Pregnant-S2	Pregnant-S1	1.03	0.004
OTU 91	*Acetitomaculum*	Pregnant-S2 vs Non-Pregnant-S2	Pregnant-S2	2.46	0.004
OTU 99	*Paeniglutamicibacter*	Pregnant-S1 vs Non-Pregnant-S1	Non-Pregnant-S1	1.22	0.002
OTU 99	*Paeniglutamicibacter*	Pregnant-S2 vs Non-Pregnant-S1	Pregnant-S2	3.99	0.002
OTU 99	*Paeniglutamicibacter*	Pregnant-S1 vs Non-Pregnant-S2	Non-Pregnant-S2	3.69	0.002

OTU 99	*Paeniglutamicibacter*	Pregnant-S2 vs Non-Pregnant-S2	Pregnant-S2	1.53	0.002

Supplementary Table S8. Alpha diversity comparison results when using Eq. 1 to determine differences in vaginal microbiota before and during pregnancy (S1, S2 and S3). Including significant pairwise comparisons.

Chao Species Richness

Type 3 Tests of Fixed Effects

Effect	Num DF	Den DF	F Value	Pr > F
sampling	2	50	2.96	0.06

Simpson Evenness

Type 3 Tests of Fixed Effects

Effect	Num DF	Den DF	F Value	Pr > F
sampling	2	50	37.15	<0.001

Significant pairwise comparisons of Simson Evenness across levels of fixed effects

Effect	Pairs			Estimate	SE	DF	t Value	P value	Adjustment	Q value
sampling	S1	vs	S2	-0.3012	0.03527	50	-8.54	<0.001	Tukey-Kramer	<0.001
sampling	S1	vs	S3	-0.2651	0.03623	50	-7.32	<0.001	Tukey-Kramer	<0.001
sampling	S2	vs	S3	0.03614	0.02426	50	1.49	0.143	Tukey-Kramer	0.304

Shannon Diversity

Type 3 Tests of Fixed Effects

Effect	Num DF	Den DF	F Value	Pr > F
sampling	2	50	37.87	<0.001

Significant pairwise comparisons of Shannon Diversity estimates across levels of fixed effects

Effect	Pairs			Estimate	SE	DF	t Value	P value	Adjustment	Q value
sampling	S1	vs	S2	-2.2697	0.2624	50	-8.65	<0.001	Tukey-Kramer	<0.001
sampling	S1	vs	S3	-1.5456	0.2756	50	-5.61	<0.001	Tukey-Kramer	<0.001
sampling	S2	vs	S3	0.7241	0.2002	50	3.62	<0.001	Tukey-Kramer	0.002

Supplementary Table S9. PERMANOVA and BetaDisperser results when using Eq. 2 to determine differences in vaginal microbiota of pregnant animals before and during pregnancy (S1, S2 and S3).

PERMANOVA

adonis(formula = data_bray ~ sampling, data = data_sub_type_df)

Permutation: free

Number of permutations: 999

	Df	SumsOfSqs	MeanSqs	F.Model	R2	Pr(>F)
sampling	2	7.268	3.6338	12.237	0.1488	0.001
Residuals	140	41.573	0.297		0.8512	
Total	142	48.841			1	

Betadisperser

Permutation test for homogeneity of multivariate dispersions

Permutation: free

Number of permutations: 999

Response: Distances

	Df	Sum Sq	Mean Sq	F	N.Perm	Pr(>F)
Groups	2	0.35806	0.179031	17.257	999	0.001
Residuals	140	1.4524	0.010374			

Supplementary Table S10. Differences in OTUs between vaginal microbiota of ewes with successful pregnancy outcome before and during pregnancy (S1, S2 and S3). OTUs are significantly different based on fixed effect of time point.

OTU	Taxonomy (Silva v138)	Comparison	More abundant in	Log2FC	Q- value
OTU 1	*Ureaplasma*	S1-S2	S1	1.13	0.002
OTU 1	*Ureaplasma*	S2-S3	S3	0.82	0.002
OTU 2	unclassified *Pasteurellaceae*	S1-S2	S1	1.84	<0.001
OTU 2	unclassified *Pasteurellaceae*	S1-S3	S1	2.48	<0.001
OTU 4	*Escherichia-Shigella*	S1-S2	S2	0.71	<0.001
OTU 4	*Escherichia-Shigella*	S1-S3	S3	0.49	<0.001
OTU 5	*Histophilus*	S1-S2	S1	2.78	<0.001
OTU 5	*Histophilus*	S1-S3	S1	5.67	<0.001
OTU 6	*Streptococcus*	S1-S2	S2	1.29	<0.001
OTU 6	*Streptococcus*	S1-S3	S3	1.26	<0.001
OTU 7	*Streptococcus*	S1-S2	S2	1.12	<0.001
OTU 7	*Streptococcus*	S1-S3	S3	0.95	<0.001
OTU 8	*Oscillospiraceae* UCG-005	S1-S2	S2	0.49	0.002
OTU 8	*Oscillospiraceae* UCG-005	S2-S3	S2	0.68	0.002
OTU 9	*Oscillospiraceae* UCG-005	S1-S2	S2	0.56	0.001
OTU 9	*Oscillospiraceae* UCG-005	S2-S3	S2	0.62	0.001
OTU 10	*Staphylococcus*	S1-S2	S1	1.19	0.034
OTU 11	*Corynebacterium*	S1-S2	S2	1.23	<0.001
OTU 11	*Corynebacterium*	S1-S3	S3	1.35	<0.001
OTU 12	*Staphylococcus*	S1-S2	S2	1.92	<0.001
OTU 12	*Staphylococcus*	S1-S3	S3	2.19	<0.001
OTU 13	*Jeotgalicoccus*	S1-S2	S2	0.52	<0.001
OTU 13	*Jeotgalicoccus*	S1-S3	S3	1.49	<0.001
OTU 13	*Jeotgalicoccus*	S2-S3	S3	0.97	<0.001

OTU 14	*Jeotgalicoccus*	S1-S2	S2	2.17	<0.001
OTU 14	*Jeotgalicoccus*	S1-S3	S3	1.23	<0.001
OTU 14	*Jeotgalicoccus*	S2-S3	S2	0.94	<0.001
OTU 15	unclassified *Leptotrichiaceae*	S1-S2	S1	3.25	<0.001
OTU 15	unclassified *Leptotrichiaceae*	S1-S3	S1	3.29	<0.001
OTU 16	*Leptotrichia*	S1-S2	S1	2.53	<0.001
OTU 16	*Leptotrichia*	S1-S3	S1	7.45	<0.001
OTU 16	*Leptotrichia*	S2-S3	S2	4.92	<0.001
OTU 17	*Romboutsia*	S1-S2	S2	0.95	<0.001
OTU 17	*Romboutsia*	S1-S3	S3	0.94	<0.001
OTU 18	unclassified *Aerococcaceae*	S1-S2	S1	4.56	<0.001
OTU 18	unclassified *Aerococcaceae*	S1-S3	S1	1.19	<0.001
OTU 18	unclassified *Aerococcaceae*	S2-S3	S3	3.37	<0.001
OTU 20	*Psychrobacter*	S1-S2	S2	5.98	<0.001
OTU 20	*Psychrobacter*	S1-S3	S3	5.7	<0.001
OTU 21	*Turicibacter*	S1-S2	S2	2.55	<0.001
OTU 21	*Turicibacter*	S1-S3	S3	2.6	<0.001
OTU 22	*Mannheimia*	S1-S2	S1	4.66	<0.001
OTU 22	*Mannheimia*	S1-S3	S1	8.42	<0.001
OTU 22	*Mannheimia*	S2-S3	S2	3.77	<0.001
OTU 23	*Oscillospiraceae* UCG-005	S1-S2	S2	0.7	<0.001
OTU 23	*Oscillospiraceae* UCG-005	S1-S3	S3	0.83	<0.001
OTU 24	*Psychrobacter*	S1-S2	S2	6.32	<0.001
OTU 24	*Psychrobacter*	S1-S3	S3	6.21	<0.001
OTU 26	unclassified *Peptostreptococcaceae*	S1-S2	S2	1.23	<0.001
OTU 26	unclassified *Peptostreptococcaceae*	S1-S3	S3	0.99	<0.001
OTU 27	*Staphylococcus*	S1-S2	S2	1.8	<0.001

OTU 27	*Staphylococcus*	S1-S3	S3	2.44	<0.001
OTU 27	*Staphylococcus*	S2-S3	S3	0.64	<0.001
OTU 28	*Treponema*	S1-S2	S2	1.41	<0.001
OTU 28	*Treponema*	S1-S3	S3	0.91	<0.001
OTU 30	*Methanobrevibacter*	S1-S2	S2	0.84	<0.001
OTU 30	*Methanobrevibacter*	S1-S3	S3	0.72	<0.001
OTU 31	*Oscillospiraceae* UCG-005	S1-S2	S2	0.49	<0.001
OTU 31	*Oscillospiraceae* UCG-005	S1-S3	S1	0.43	<0.001
OTU 31	*Oscillospiraceae* UCG-005	S2-S3	S2	0.92	<0.001
OTU 32	*Acinetobacter*	S1-S2	S1	3.42	<0.001
OTU 32	*Acinetobacter*	S1-S3	S1	4.32	<0.001
OTU 33	*Bacteroides*	S1-S2	S2	0.78	<0.001
OTU 33	*Bacteroides*	S1-S3	S3	0.44	<0.001
OTU 33	*Bacteroides*	S2-S3	S2	0.34	<0.001
OTU 34	*Anaerococcus*	S1-S2	S2	0.83	<0.001
OTU 34	*Anaerococcus*	S1-S3	S3	1.5	<0.001
OTU 34	*Anaerococcus*	S2-S3	S3	0.67	<0.001
OTU 35	*Bifidobacterium*	S1-S2	S2	4.4	<0.001
OTU 35	*Bifidobacterium*	S1-S3	S3	4.2	<0.001
OTU 36	unclassified *Staphylococcaceae*	S1-S2	S2	1.36	<0.001
OTU 36	unclassified *Staphylococcaceae*	S1-S3	S3	0.96	<0.001
OTU 37	*Turicibacter*	S1-S2	S2	1.13	<0.001
OTU 37	*Turicibacter*	S1-S3	S3	1	<0.001
OTU 39	*Prevotellaceae* UCG-003	S1-S2	S2	0.64	0.001
OTU 39	*Prevotellaceae UCG-003*	S1-S3	S3	0.43	0.001
OTU 40	*Clostridium* sensu stricto 1	S1-S2	S2	3.14	<0.001
OTU 40	*Clostridium* sensu stricto 1	S1-S3	S3	3.07	<0.001

OTU 41	*Alistipes*	S1-S2	S2	0.5	<0.001
OTU 41	*Alistipes*	S1-S3	S1	1.06	<0.001
OTU 41	*Alistipes*	S2-S3	S2	1.56	<0.001
OTU 42	unclassified *Planococcaceae*	S1-S2	S2	0.59	<0.001
OTU 42	unclassified *Planococcaceae*	S1-S3	S1	1.52	<0.001
OTU 42	unclassified *Planococcaceae*	S2-S3	S2	2.11	<0.001
OTU 43	*Oscillospiraceae* UCG-005	S1-S2	S2	0.41	0.032
OTU 44	*Paeniclostridium*	S1-S2	S2	1.62	<0.001
OTU 44	*Paeniclostridium*	S1-S3	S3	1.34	<0.001
OTU 45	*Methanobrevibacter*	S1-S2	S2	0.85	<0.001
OTU 45	*Methanobrevibacter*	S2-S3	S2	0.79	<0.001
OTU 46	*Glutamicibacter*	S1-S2	S2	7.99	<0.001
OTU 46	*Glutamicibacter*	S1-S3	S3	10.68	<0.001
OTU 46	*Glutamicibacter*	S2-S3	S3	2.69	<0.001
OTU 47	*Treponema*	S1-S2	S2	2.03	<0.001
OTU 47	*Treponema*	S2-S3	S2	1.1	<0.001
OTU 48	*Planococcus*	S1-S2	S2	1.58	<0.001
OTU 48	*Planococcus*	S1-S3	S3	1.03	<0.001
OTU 48	*Planococcus*	S2-S3	S2	0.56	<0.001
OTU 49	unclassified *Lachnospiraceae*	S1-S2	S2	2.44	<0.001
OTU 49	unclassified *Lachnospiraceae*	S1-S3	S3	2.12	<0.001
OTU 50	*Oscillospiraceae* UCG-005	S1-S2	S2	0.68	<0.001
OTU 50	*Oscillospiraceae UCG-005*	S1-S3	S3	0.56	<0.001
OTU 51	*Oscillospiraceae* UCG-005	S1-S2	S2	0.67	0.001
OTU 51	*Oscillospiraceae* UCG-005	S2-S3	S2	0.38	0.001
OTU 53	*Brachybacterium*	S1-S2	S2	1.89	<0.001
OTU 53	*Brachybacterium*	S1-S3	S3	1.94	<0.001

OTU 54	unclassified *Bacillales*	S1-S2	S2	3.8	0.025
OTU 55	*Methanobrevibacter*	S1-S2	S2	1.12	<0.001
OTU 55	*Methanobrevibacter*	S1-S3	S3	2.15	<0.001
OTU 55	*Methanobrevibacter*	S2-S3	S3	1.03	<0.001
OTU 56	*Micrococcus*	S1-S2	S1	1.19	<0.001
OTU 56	*Micrococcus*	S1-S3	S1	2.21	<0.001
OTU 56	*Micrococcus*	S2-S3	S2	1.02	<0.001
OTU 57	*Brevibacterium*	S1-S2	S2	1.71	<0.001
OTU 57	*Brevibacterium*	S1-S3	S3	0.86	<0.001
OTU 57	*Brevibacterium*	S2-S3	S2	0.85	<0.001
OTU 58	*Streptococcus*	S1-S3	S3	4.5	<0.001
OTU 58	*Streptococcus*	S2-S3	S3	7.51	<0.001
OTU 59	*Alistipes*	S1-S2	S2	1.17	<0.001
OTU 59	*Alistipes*	S1-S3	S3	0.67	<0.001
OTU 59	*Alistipes*	S2-S3	S2	0.5	<0.001
OTU 60	unclassified *Lactobacillales*	S1-S2	S1	2.08	<0.001
OTU 61	*Psychrobacter*	S1-S2	S2	6.04	<0.001
OTU 61	*Psychrobacter*	S1-S3	S3	5.57	<0.001
OTU 62	*Facklamia*	S1-S2	S2	5.76	<0.001
OTU 62	*Facklamia*	S1-S3	S3	5.76	<0.001
OTU 63	*Campylobacter*	S1-S2	S2	0.8	<0.001
OTU 63	*Campylobacter*	S2-S3	S2	0.64	<0.001
OTU 64	*Mogibacterium*	S1-S2	S2	0.66	0.001
OTU 64	*Mogibacterium*	S2-S3	S2	0.54	0.001
OTU 65	unclassified *Gammaproteobacteria*	S1-S2	S2	5.71	<0.001
OTU 65	unclassified *Gammaproteobacteria*	S1-S3	S3	3.63	<0.001
OTU 65	unclassified *Gammaproteobacteria*	S2-S3	S2	2.07	<0.001

OTU 66	*Bergeyella*	S1-S2	S1	6.03	<0.001
OTU 66	*Bergeyella*	S1-S3	S1	6.07	<0.001
OTU 67	unclassified *Lactobacillales*	S1-S2	S2	7.2	<0.001
OTU 67	unclassified *Lactobacillales*	S1-S3	S3	7	<0.001
OTU 69	unclassified *Ruminococcaceae*	S1-S2	S2	5.19	<0.001
OTU 69	unclassified *Ruminococcaceae*	S1-S3	S3	4.6	<0.001
OTU 70	*Prevotellaceae* UCG-003	S1-S2	S2	1.44	<0.001
OTU 70	*Prevotellaceae* UCG-003	S2-S3	S2	1.86	<0.001
OTU 71	*Corynebacterium*	S1-S2	S1	1.03	<0.001
OTU 71	*Corynebacterium*	S1-S3	S1	1.94	<0.001
OTU 71	*Corynebacterium*	S2-S3	S2	0.91	<0.001
OTU 72	*Romboutsia*	S1-S2	S2	1.5	<0.001
OTU 72	*Romboutsia*	S1-S3	S3	1.23	<0.001
OTU 73	unclassified *Bacteroidales*	S1-S2	S2	0.92	<0.001
OTU 73	unclassified *Bacteroidales*	S2-S3	S2	1.25	<0.001
OTU 74	*Treponema*	S1-S2	S2	4.76	<0.001
OTU 74	*Treponema*	S1-S3	S3	4.91	<0.001
OTU 75	*Glutamicibacter*	S1-S2	S2	5.29	<0.001
OTU 75	*Glutamicibacter*	S2-S3	S2	4.43	<0.001
OTU 76	*Salinicoccus*	S1-S2	S1	2.94	<0.001
OTU 76	*Salinicoccus*	S1-S3	S1	3.75	<0.001
OTU 77	*Corynebacterium*	S1-S2	S2	10.56	<0.001
OTU 77	*Corynebacterium*	S1-S3	S3	7.93	<0.001
OTU 77	*Corynebacterium*	S2-S3	S2	2.63	<0.001
OTU 78	*Succinivibrio*	S1-S3	S3	5.25	<0.001
OTU 78	*Succinivibrio*	S2-S3	S3	3.62	<0.001
OTU 81	*Corynebacterium*	S1-S2	S1	1.17	0.003

OTU 82	*Corynebacterium*	S1-S2	S2	1.05	0.008
OTU 82	*Corynebacterium*	S1-S3	S3	0.78	0.008
OTU 83	*Finegoldia*	S1-S2	S2	1.36	0.002
OTU 83	*Finegoldia*	S1-S3	S3	2.34	0.002
OTU 84	*Campylobacter*	S1-S2	S1	3.37	0.012
OTU 84	*Campylobacter*	S2-S3	S3	4.77	0.012
OTU 85	*Kocuria*	S1-S2	S1	1.7	<0.001
OTU 85	*Kocuria*	S1-S3	S1	2.75	<0.001
OTU 86	*Christensenellaceae R-7 group*	S1-S3	S1	1.79	<0.001
OTU 86	*Christensenellaceae* R-7 group	S2-S3	S2	1.91	<0.001
OTU 87	*Fusobacterium*	S1-S2	S1	4.99	<0.001
OTU 87	*Fusobacterium*	S1-S3	S3	2.07	<0.001
OTU 87	*Fusobacterium*	S2-S3	S3	7.06	<0.001
OTU 88	*Lachnospiraceae* NK3A20 group	S1-S2	S2	0.8	<0.001
OTU 88	*Lachnospiraceae NK3A20 group*	S1-S3	S3	0.48	<0.001
OTU 89	*Anaerococcus*	S1-S3	S3	1.79	0.006
OTU 89	*Anaerococcus*	S2-S3	S3	2.67	0.006
OTU 90	unclassified *Ruminococcaceae*	S1-S2	S2	3.01	<0.001
OTU 90	unclassified *Ruminococcaceae*	S1-S3	S3	2.49	<0.001
OTU 91	*Acetitomaculum*	S1-S2	S2	1.42	<0.001
OTU 91	*Acetitomaculum*	S1-S3	S3	0.8	<0.001
OTU 91	*Acetitomaculum*	S2-S3	S2	0.62	<0.001
OTU 92	unclassified *Planococcaceae*	S1-S2	S1	1.33	0.001
OTU 92	unclassified *Planococcaceae*	S1-S3	S1	1.3	0.001
OTU 93	*Treponema*	S1-S2	S2	4.1	0.002
OTU 93	*Treponema*	S2-S3	S2	1.49	0.002
OTU 94	*Phascolarctobacterium*	S1-S2	S2	0.73	0.001

OTU 94	*Phascolarctobacterium*	S2-S3	S2	1.06	0.001
OTU 95	*Bacteroidales* RF16 group ge	S1-S2	S2	1.02	<0.001
OTU 95	*Bacteroidales* RF16 group ge	S1-S3	S3	0.47	<0.001
OTU 95	*Bacteroidales* RF16 group ge	S2-S3	S2	0.55	<0.001
OTU 96	*Oscillospirales* ge	S1-S2	S2	1.08	<0.001
OTU 96	*Oscillospirales* ge	S1-S3	S3	0.55	<0.001
OTU 96	*Oscillospirales* ge	S2-S3	S2	0.53	<0.001
OTU 97	*Oscillospiraceae* UCG-005	S1-S2	S2	0.67	<0.001
OTU 97	*Oscillospiraceae* UCG-005	S1-S3	S3	1.07	<0.001
OTU 99	*Paeniglutamicibacter*	S1-S2	S2	6.04	<0.001
OTU 99	*Paeniglutamicibacter*	S1-S3	S3	6.76	<0.001
OTU 100	Uncultured *Paludibacteraceae*	S1-S3	S1	1.31	0.001
OTU 100	Uncultured *Paludibacteraceae*	S2-S3	S2	1.75	0.001
